# Genome-wide identification, phylogeny, evolution, and expression patterns of MtN3/saliva/SWEET genes and functional analysis of *BcNS* in *Brassica rapa*

**DOI:** 10.1186/s12864-018-4554-8

**Published:** 2018-03-02

**Authors:** Liming Miao, Yanxia Lv, Lijun Kong, Qizhen Chen, Chaoquan Chen, Jia Li, Fanhuan Zeng, Shenyun Wang, Jianbin Li, Li Huang, Jiashu Cao, Xiaolin Yu

**Affiliations:** 1 Laboratory of Cell and Molecular Biology, Institute of Vegetable Science, Zhejiang University, 866 Yuhangtang Road, Zhejiang Province, Hangzhou, 310058 P. R. China; 2 Laboratory of Horticultural Plant Growth and Quality Regulation, Ministry of Agriculture, Zhejiang Province, Hangzhou, 310058 P. R. China; 3 Key Laboratory of Horticultural Plant Integrative Biology, Zhejiang Province, Hangzhou, 310058 P. R. China; 4 Institute of Vegetable Science, Jiangsu Academy of Agricultural Sciences, Nanjing, 210014 P. R. China; 5 Jiangsu Key Laboratory for Horticulture Crop Genetic Improvement, Nanjing, 210014 P. R. China

**Keywords:** Chinese cabbage (*Brassica rapa * syn. *Brassica campestris*), MtN3/saliva/SWEETs, Gene family, *BcNS*, Nectary, Functional analysis

## Abstract

**Background:**

Members of the MtN3/saliva/SWEET gene family are present in various organisms and are highly conserved. Their precise biochemical functions remain unclear, especially in Chinese cabbage. Based on the whole genome sequence, this study aims to identify the MtN3/saliva/SWEETs family members in Chinese cabbage and to analyze their classification, gene structure, chromosome distribution, phylogenetic relationship, expression pattern, and biological functions.

**Results:**

We identified 34 SWEET genes in Chinese cabbage and analyzed their localization on chromosomes and transmembrane regions of their corresponding proteins. Analysis of a phylogenetic tree indicated that there were at least 17 supposed ancestor genes before the separation in *Brassica rapa* and *Arabidopsis*. The expression patterns of these genes in different tissues and flower developmental stages of Chinese cabbage showed that they are mainly involved in reproductive development. The Ka/Ks ratio between paralogous SWEET gene pairs of *B. rapa* were far less than 1. In our previous study, *At2g39060* homologous gene *Bra000116* (*BraSWEET9*, also named *BcNS*, *Brassica* Nectary and Stamen) played an important role during flower development in Chinese cabbage. Instantaneous expression experiments in onion epidermal cells showed that the gene encoding this protein is localized to the plasma membrane. A basal nectary split is the phenotype of transgenic plants transformed with the antisense expression vector.

**Conclusion:**

This study is the first to perform a sequence analysis, structures analysis, physiological and biochemical characteristics analysis of the MtN3/saliva/SWEETs gene in Chinese cabbage and to verify the function of *BcNS*. A total of 34 SWEET genes were identified and they are distributed among ten chromosomes and one scaffold. The Ka/Ks ratio implies that the duplication genes suffered strong purifying selection for retention. These genes were differentially expressed in different floral organs. The phenotypes of the transgenic plants indicated that *BcNs* participates in the development of the floral nectary. This study provides a basis for further functional analysis of the MtN3/saliva/SWEETs gene family.

**Electronic supplementary material:**

The online version of this article (10.1186/s12864-018-4554-8) contains supplementary material, which is available to authorized users.

## Background

The MtN3/saliva/SWEET gene family widely exists in high-level eukaryotes and can be found in protozoa, metazoa, fungi, bacteria, and archaea [1, 2]. The first gene of this family was identified to be *MtN3* (*Medicago truncatula* Nodulin3), which is a root-nodule-related gene in *M. truncatula* and is associated with *Rhizobium*-induced nodule development [3]. Thereafter, a homologous gene of *MtN3* was found in the saliva of *Drosophila*, and this gene is expressed in the salivary gland during embryonic development [4]. Since then, proteins with the conserved domains have been found in mice, humans, sea squirts, petunia, rice, *Arabidopsis*, and other plants. The conserved transmembrane domain is named as the MtN3/saliva domain; proteins containing this domain are assigned to the MtN3/saliva protein family [2]. A new family of sugar carriers that use sugar optical sensors have been found and named the SWEET family [5]. SWEET and the previously reported MtN3/saliva gene family belong to the same family [6]. MtN3/saliva/SWEET proteins can be classified into two types based on the number of MtN3/saliva domains: the first type consists of seven transmembrane helices that harbor two MtN3/saliva domains, and the second type comprises three transmembrane helices that harbor one MtN3/saliva domain [7]. These domains are highly conserved in the evolutionary process of the MtN3/saliva/SWEET gene family. The number of the members in this family differs among different species and is high in some plant species.

The evolution of the MtN3/saliva/SWEET gene family is highly conserved and these genes are involved in many different physiological functions in plants, including reproductive development, senescence, abiotic stress response, and host-pathogen interactions. The MtN3/saliva/SWEET family genes are associated with fertility in plants. *Os11N3/SWEET14*-knockout mutants exhibit reduced seed size and delayed reproductive development [8]. *NEC1*, an MtN3/saliva/SWEET gene, is specifically expressed in the nectary tube and stamen of *Petunia* [9]. When the gene was cosuppressed, the transgenic plants showed male sterility due to failure of anther dehiscence [10]. *RPG1* (RUPTURED POLLEN GRAIN 1), also named *AtSWEET8*, is a necessary gene for anther development in *Arabidopsis* [11]; the gene was highly expressed in the tapetum and pollen mother cells from stage 5 to stage 7. The male fertility of *rpg1* decreased significantly, and most of the pollen grains were degraded during development. The microspore membrane of *rpg1* became dysfunctional in the tetrad stage, leading to sporopollenin deposition onto the microspore surface, which influenced pollen wall development and pollen fertility [11].

The MtN3/saliva/SWEET family genes are involved in senescence, abiotic stress response, and host-pathogen interactions in plants. The SAG29 (AtSWEET15) protein is located on the plasma membrane and regulates cell activity in a high-salt environment; the protein expression is induced by the ABA pathway, depending on osmotic pressure in plant senescent tissues; *SAG29* mutants are less sensitive to high salt environments, whereas *SAG29* 35S-overexpressing transgenic plants are highly sensitive to high salt environments and exhibit accelerated aging phenotypes [12]. Suppressing the expression of either the dominant or recessive allele of *Xa13/Os8N3/OsSWEET11* enhances the resistance to PXO99, which is an incompatible strain of *xa13* [13].

Cruciferous plants occupies a very important niche in agricultural production, and nectaries play vital roles in the pollination and fertilization of the plants. However, the molecular mechanisms involved in nectary development are very limited. *CRABS CLAW*, *BOP1 (BLADE-ON-PETIOLE1*), *BOP2* [14, 15], and *CWINV4* (*CELL WALL INVERTASE 4*) were reported which involved in nectary development*.* In *cwinv4* mutant plants, the nectary cannot secrete honeydew [16, 17]*.* There is a study that shows that many genes are involved in photosynthesis in turnips [18]. The another study assumed that nectaries could make carbohydrates themselves through photosynthesis [19]. The nectaries of cruciferous plants are small, and their structure is complicated, so they are difficulty to be studied. However, exploring nectary development will benefit our understanding of the plants’ reproductive process.

So far, the MtN3/saliva/SWEET gene family has been rarely reported in plants, and the molecular mechanism of the gene family remains poorly understood. This study aims to clarify the biological function of MtN3/saliva/SWEET family genes in Chinese cabbage. Thirty four genes belonging to the MtN3/saliva/SWEET family were identified in cabbage. Gene and protein sequences were evaluated and compared by analyzing the protein sequences, evolution of the gene family, protein transmembrane domain, gene chromosomal localization, and gene structure. The expression characteristics of MtN3/saliva/SWEET family genes were studied in different tissues and flower developmental stages to provide some clues of their function.

## Methods

### Identification and isolation of MtN3/saliva/SWEET family genes in *Brassica rapa*

To identify MtN3/saliva/SWEET family genes, we found candidate gene sequences with the reported MtN3/saliva/SWEET family genes in the *Arabidopsis* Genome Database TAIR (http://www.arabidopsis.org/), and then verified the obtained sequences in the conserved domain database of NCBI (National Center for Biotechnology Information) (http: //www.ncbi. nlm.nih.gov/Structure/cdd/wrpsb.cgi). Ultimately, 17 Arabidopsis MtN3/saliva/SWEET family members were found. Then, we obtained the candidate genes of the MtN3/saliva/SWEET family in the *Brassica* database (http://brassicadb.org/brad/searchGene.php) by analyzing *Arabidopsis* MtN3/saliva/SWEET family members’ amino-acid sequences and domain sequences (Additional file 1: Table S1) with BLASTP. The Pfam database (E-value is 1.0) (http://pfam.janelia.org/) and the Conserved Domain Database (CDD) of NCBI were used to determine whether each of the sequences each harbored one or more MtN3/saliva/SWEET conserved domains (MtN3_slv, PF03083.15). The genes that did not contain the known conserved domains of the gene families were excluded from further analysis. Eventually, we identified 34 genes of the MtN3/saliva/SWEET family genes in Chinese cabbage.

### Chromosomal localization of the MtN3/saliva/SWEET genes

We searched the start location and stop location on the *B. rapa* chromosomes of all MtN3/saliva/SWEET family genes in the *Brassica* database. Chromosomal localization of these genes was performed by MapChart software based on the relative location of each gene on each chromosome [20]. Tandem duplications were defined as genes located within 20 loci from each other [21, 22].

### Multiple sequence alignment and phylogenetic analysis of MtN3/saliva/SWEET family genes

We downloaded the full-length DNA sequences and the cDNA sequences of MtN3/saliva/SWEETs genes from the *Brassica* database. GSDS (Gene Structure Display Server) (http://gsds.cbi.pku.edu.cn/index.php) was utilized to analyze the locations of introns and exons. Homologous sequence alignment of the 34 MtN3/saliva/SWEETs family genes was performed with ClustalW [23], and then the sequence alignment results were considered the basis for generating the unrooted phylogenetic tree of the MtN3/saliva/SWEET family members with MEGA (version 5.0) [24]. All parameters used were default parameters. Phylogenetic trees were generated with the value of the 1000 bootstrap samples by the neighbor-joining (NJ) method [24].

### Nonsynonymous substitution rate and synonymous substitution rate between orthologous SWEET genes between *A. thaliana* and *B. rapa*

Synteny analysis of MtN3/saliva/SWEETs genes between were *A. thaliana* and *B. rapa* performed with PGDD (http://chibba.agtec.uga.edu/duplication/). The occurrence of duplication events and homologous genes divergence, as well as the selective pressure on duplicated genes, were estimated by calculating synonymous (*K*_s_) and non-synonymous substitutions (*K*_a_) per site between the orthologous genes using the LOCUS SEARCH utility of PGDD. The divergence time was calculated using the neutral substitution rate of 1.5 × 10^− 8^ substitutions per site per year for the chalcone synthase gene (*Chs*) [25].

### Conserved short amino-acid sequence analysis of MtN3/saliva/SWEET in *B. rapa*

We used MEME to analyze the common conservative motifs of 34 MtN3/saliva/SWEETs amino-acid sequences from Chinese cabbage [26]. Phylogenetic tree was generated with the value of 1000 bootstrap samples by the NJ method.

### Transmembrane helices and hydrophobicity analysis of the MtN3/saliva/SWEET family proteins in *B. rapa*

The transmembrane helices of the MtN3/saliva/SWEET family proteins were predicted by the TMHMM (http://www.cbs.dtu.dk/services/TMHMM/) with their amino acid sequences [27]. Hydrophobicity analysis of MtN3/saliva/SWEET family proteins were analyzed with Protscale (http://web.expasy.org/protscale/) in accordance with the scoring criteria of Hphob/Kyte and Doolittle [28]. The GRAVY value for a peptide or protein was calculated as the sum of hydropathy values of all the amino acids divided by the number of residues in the sequence. A larger positive hydrophilic value indicated stronger hydrophobicity. By contrast, a smaller negative hydrophilic value indicated stronger hydrophilicity. Hydrophilic values taht ranged from + 0.5 to − 0.5 mainly corresponded to amphoteric amino acids. Furthermore, the isoelectric points (pIs) and molecular weights of the MtN3/saliva/SWEET proteins were predicted using ExPASy (http://web.expasy.org/ compute_pi/) [29].

### *Cis*-element analysis of the promoter sequences of MtN3/saliva/SWEET family genes in *B. rapa*

To identify the *cis*-elements in the promoter sequences of the 34 MtN3/saliva/SWEET family genes, the sequences of these genes were used as query sequences for the BLASTN search against the *Brassica* database. A 1500 bp of genomic sequences upstream of the start codon of each MtN3/saliva/SWEET family gene was chosen. Afterward, PlantCare (http://bioinformatics.psb.ugent.be/webtools/plantcare/html/) and a manual search were employed to predict the *cis*-elements [30].

### Quantitative reverse-transcription PCR (qRT-PCR) analysis of the MtN3/saliva/SWEET family genes in different tissues or different floral organs

‘Chiifu-401’ is the species that was used in genome sequencing, so ‘Chiifu-401’ was used in our study for qRT-PCR analysis and grown in the experimental base of our laboratory. We then obtained roots, stems, leaves, flowers, pods, sepals, petals, stamens, pistils, nectar, big buds (> 2.0 mm), and small buds (< 2.0 mm) in the flowering period. All the samples were collected and immediately frozen in liquid nitrogen and stored at − 80 °C.

Total RNA was extracted from previous materials using the TRIzol reagent (Invitrogen, USA) in accordance with the manufacturer’s instructions. The first cDNA strand was generated following the manufacturer’s protocol using the Takara Reverse Transcription System (Japan). qRT-PCR was performed using the primer pairs listed in Additional file 2: Table S2. The primers were designed using the Primer program (version 5.0). The specificity of each primer to its corresponding gene was checked using the BLASTN program of the NCBI. A sample of cDNA (0.2 μg) was subjected to real-time PCR in a final volume of 20 μL containing 10 μL of SYBR Green Master Mix Reagent (Takara, Japan) and 0.8 μL of specific primers. Three biological and three technical replicates for each sample were performed in a real-time PCR machine (Bio-Rad CFX Manager). The heating program was as follows: 95 °C for 30 s, followed by 95 °C at 10 s for 40 cycles, and 52 °C at 30 s (varying with specific primers). To normalize the total amount of cDNA present in each reaction, the Chinese cabbage *Cyp* gene was amplified as an endogenous control for calibrating relative expression. The 2 − ∆∆CT method of relative gene quantification recommended by Applied Biosystems (PE Applied Biosystems, USA) was used to calculate the expression level of different treatments [31].

In our previous study, we found that *BcNS* plays an important role in Chinese cabbage, so we studied the expression pattern of *BcNS* here. The expression in open flowers, I ~ V buds (I: < 1 mm, II: 1.2 ~ 1.6 mm, III: 1.8 ~ 2.2 mm, IV: 2.4 ~ 2.8 mm, V: 3.0 ~ 3.4 mm), tender pods, stems, leaves and pistils in ‘*Bajh97-01A/B*’ sterile and fertile plants were examined 1 h, 8 h, and 24 h after pollinating with semi quantitative PCR, and the gene expression in sepals, petals, stamen, pistils and nectaries in ‘*Bajh97-01A/B*’ sterile and fertile plants were detected with semi-quantitative PCR and quantitative reverse-transcription PCR.

### In situ hybridization

Flower buds at different developmental stages were fixed in 4% formaldehyde phosphate-buffered saline (PBS) solution with 0.1% Triton X-100 and 0.1% Tween-20, serially dehydrated, cleared with dimethyl benzene, and embedded in paraffin. Sections (8 μm) of flower buds were hybridized to specific digoxigenin-labelled RNA probes (Roche). Templates for the BcNS-specific probes were obtained from amplification with specific primers (upstream primer: 5’-TACGGATCCAGTCTCGCCGTCTTTGCT-3′; downstream primer: 5’-CGCTCTAGACCGTGGTGCTTGAGTTTG-3′; the underlined section is the enzyme cut site). The sense and antisense probes were synthesized using an SP6/ T7 transcription kit (Roche) [32].

### Subcellular localization of *BcNS* in the onion epidermal cell

We studied the subcellular localization of BcNS in the onion epidermal cell. First, the *BcNS* gene fragment was amplified from the nectaries of Chinese cabbage ‘Chiifu-401’ with a high-fidelity enzyme (TOYOBO, Japan). To overexpress the YFP (Yellow Fluorescent Protein) marker protein of the pB7YWG2.0-YFP, the stop codon of *BcNS* had to be replaced. Otherwise, the 5′-end of the upstream primer should add “CACC” when using the TOPO isomerase to connect to the entry vector. Thus, we designed the primer as follows: upstream (BcNS-GF), 5′-CACCATGGTGTTCATCAAAGTTCATCAA-3′; downstream (BcNS-GR), 5′-GTAAGCCGTGGTGCTTGAGT-3′. The PCR reaction was programmed to heat the samples at 94 °C for 30 min, followed by 94 °C for 30 s in 29 cycles, 52 °C for 30 s (varying with specific primers), 68 °C for 90 s, and 68 °C extended to 7 min. The verified sequence was connect to the entry vector using the pENTR™ Directional TOPO® Cloning Kit (Invitrogen, USA). The target sequence was replaced into the expression vector through homologous replacement with the Gateway®LR Clonase™ Enzyme Mix (Invitrogen, USA). The constructed vector was transferred into an onion epidermal cell for transient expression by a gene gun.

### Construction of the antisense expression vector of *BcNS* and molecular detection in transgenic plants

Flowering Chinese cabbage is a spring variety and can normally flower without vernalization. Using this species as the experimental material can improve the experimental efficiency. Our previous study found a 99.4% similarity of *BcNS* between *B. campestris* and *B. rapa.* Therefore, flowering Chinese cabbage is a suitable material for the study of *BcNS* function. We constructed the antisense expression vector *pCAMIA1301-BcNS* of the *BcNS* gene by enzyme digestion and other routine molecular biological methods. We then introduced the vector into *Agrobacterium* LBA4404 and EHA105 for genetic transformation.

One or two leaves were obtained from the transgenic plants and the controls. Dust was wiped away with 75% alcohol, and then DNA was extracted from the samples using the CTAB (Hexadecyltrimethylammonium bromide) method for PCR testing. The leaves and calluses of the transgenic plants and negative control were placed at X-Gluc solution at 37 °C for 24–36 h. Subsequently, the samples were placed at 95% ethanol for the dehydration treatment. Finally, sample staining was observed under a microscope. The morphological characteristics of the transgenic plants were observed under an MZ16 FA Leica microscope.

### Scanning electron microscopy observations

For the scanning electron microscopy of the nectaries in transgenic plants, we followed the protocol described by the Center of Electron Microscopy, Zhejiang University. Firstly, the nectaries of the transgenic plants and their control were fixed with glutaraldehyde in 4 °C overnight. Secondly, the sample was washed with phosphate buffer three times, each time for 15 min. Then, the sample was serially dehydrated in ethanol at different concentrations for 15 min at each concentration. The last two steps used 100% ethanol, and each step lasted 20 min. The samples were dried through the critical point drying method, and then the samples were pasted onto the metal sample table with a special double-sided adhesive. After coating the samples with a metallic membrane, the samples were placed on a desktop scanning electron microscope (TM-1000) to observe the nectary and stomas.

## Results

### Identification of MtN3/saliva/SWEET family genes in *B. rapa*

Thirty-four MtN3/saliva/SWEETs genes were obtained by BLASTP against the *Arabidopsis* Genome Database TAIR, NCBI databases and *Brassica* database. These genes were then named after their *A. thaliana* homologs (Table 1). Duplication clusters were identified and found to be *BrSWEET1a/1b*, *BrSWEET2a/2b, BrSWEET3a/3b, BrSWEET4a/4b*, *BrSWEET5a/5b*, *BrSWEET7a/7b, BrSWEET11a/11bc*, *BrSWEET12a/12b*, *BrSWEET14a/14b*, *BrSWEET15a/15bc* and *BrSWEET16a/16b* (Table 2). A total of 17 *AtSWEETs* were obtained, but homologs of *Bra012412*, which we named as *BrSWEET* 18, were not found in the annotated *AtSWEETs* (Table 1). The open reading frame (ORF) lengths of the MtN3/saliva/SWEETs genes ranged from 387 bp (*BrSWEET2c*) to 951 bp (*BrSWEET15b*). The encoded polypeptides ranged from 128 amino acids (*BrSWEET2c*) to 316 amino acids (*BrSWEET15b*), with molecular weights from 14.299 kDa (*BrSWEET2c*) to 35.814 kDa (*BrSWEET15b*). The theoretical pI ranged from 7.59 (*BrSWEET2*) to 9.62 (*BrSWEET7*), and most of the pIs were more than 8.50 except *BrSWEET*2a, *BrSWEET*3b, *BrSWEET*5b, *BrSWEET*5c and *BrSWEET*15c (Table 1). The MtN3/saliva/SWEET genes were distributed on 10 chromosomes. *BrSWEET*13 was distributed on a scanffold. A total of 31 MtN3/saliva/SWEETs members contained two conserved domains, whereas the others contained only one conserved domain (Table 1).

**Table 1:** The *MtN3/Saliva/SWEETs* gene family in *Brassica rapa* and information relevant to *Arabidopsis thaliana*

Group	*Arabidopsis thaliana*						*Brassica rapa*								
	Gene name	Accession number	ORF(bp)	Deduced polypeptide			Gene name	Accession number	Chromosome	Subgenome	ORF(bp)	Deduced polypeptide			Number of domain
				Protein length (aa)	MW(kD)	PI						Protein length (aa)	MW(kD)	PI	
I	*AtSWEET1*	AT1G21460	744	247	27.268	9.2	*BrSWEET1a*	Bra016421	8	MF1	756	251	27.818	9.27	2
							*BrSWEET1b*	Bra017916	6	LF	741	246	27.185	9.3	2
	*AtSWEET2*	AT3G14770	711	236	26.467	8.5	*BrSWEET2a*	Bra021577	1	MF1	711	236	26.567	7.59	2
							*BrSWEET2b*	Bra027314	5	LF	648	215	24.078	9.16	2
							*BrSWEET2c*	Bra027312	5	LF	387	128	14.299	9.15	1
	*AtSWEET3*	AT5G53190	792	263	29.537	9.3	*BrSWEET3a*	Bra022636	2	MF2	780	259	29.328	8.71	2
							*BrSWEET3b*	Bra003075	10	LF	579	192	21.593	7.64	1
II	*AtSWEET4*	AT3G28007	756	251	27.815	8.7	*BrSWEET4a*	Bra025317	6	LF	747	248	27.488	9.07	2
							*BrSWEET4b*	Bra033034	2	MF1	738	245	27.416	8.94	2
	*AtSWEET5*	AT5G62850	723	240	27.21	8.9	*BrSWEET5a*	Bra035879	9	MF2	723	240	27.3	9.04	2
							*BrSWEET5b*	Bra029245	2	MF1	723	240	26.971	8.15	2
							*BrSWEET5c*	Bra029244	2	MF1	723	240	26.826	8.14	2
	*AtSWEET6*	AT1G66770	786	261	28.867	9.3	*/*	/	/	/	/	/	/	/	/
	*AtSWEET7*	AT4G10850	777	258	28.531	9.2	*BrSWEET7a*	Bra035270	9	LF	750	249	27.362	9.62	2
							*BrSWEET7b*	Bra000725	3	MF1	537	178	19.581	8.63	1
	*AtSWEET8*	AT5G40260	720	239	26.886	9.3	*BrSWEET8*	Bra025595	4	LF	717	238	26.793	8.97	2
III	*AtSWEET9*	AT2G39060	777	258	28.716	9.2	*BrSWEET9*	Bra000116	3	MF2	813	270	30.125	9.18	2
	*AtSWEET10*	AT5G50790	870	289	32.763	9.3	*BrSWEET10*	Bra022761	3	MF1	870	290	33.041	9.29	2
	*AtSWEET11*	AT3G48740	870	289	31.921	9.3	*BrSWEET11a*	Bra018039	6	LF	870	289	32.068	9.18	2
							*BrSWEET11b*	Bra019560	6	MF2	858	285	31.584	9.32	2
							*BrSWEET11c*	Bra029914	1	MF1	873	290	32.343	9.04	2
	*AtSWEET12*	AT5G23660	858	285	31.487	9.1	*BrSWEET12a*	Bra009700	6	LF	867	288	31.781	9.07	2
							*BrSWEET12b*	Bra026487	9	MF2	834	277	30.494	9.13	2
	*AtSWEET13*	AT5G50800	885	294	32.504	9.4	*BrSWEET13*	Bra040489	scanfold	/	885	294	32.747	9.05	2
	*AtSWEET14*	AT4G25010	846	281	30.969	9.0	*BrSWEET14a*	Bra013854	1	LF	822	273	30.105	9.29	2
							*BrSWEET14b*	Bra010477	8	MF2	819	272	29.916	9.18	2
							*BrSWEET14c*	Bra019197	3	MF1	717	238	26.408	9.08	2
	*AtSWEET15*	AT5G13170	879	292	32.936	8.2	*BrSWEET15a*	Bra023394	2	MF2	894	297	33.156	8.26	2
							*BrSWEET15b*	Bra006185	3	MF1	951	316	35.814	8.86	2
							*BrSWEET15c*	Bra008850	10	LF	630	209	23.314	7.74	2
							*BrSWEET18*	Bra012412	7	MF2	528	175	20.093	9.38	2
IV	*AtSWEET16*	AT3G16690	693	230	25.744	5.1	*BrSWEET16a*	Bra001638	3	MF2	696	231	25.679	9.06	2
							*BrSWEET16b*	Bra021190	1	MF1	696	231	25.754	8.69	2
	*AtSWEET17*	AT4G15920	726	241	26.813	6.1	*BrSWEET17a*	Bra012752	3	MF1	723	240	26.444	8.76	2
							*BrSWEET17b*	Bra038060	8	MF2	735	244	27.311	9.01	2

**Table 2:** The Non-synonymous substitution rate and synonymous substitution rate between orthologous SWEET gene pairs between *A.thaliana* and *B. rapa*

Orthologous gene pairs			Non-synonymous substitution rate (Ka)	Synonymous substitution rate (Ks)	Ka/Ks
AT1G21460	Bra016421	*BrSWEET1a*	0.0400	0.4300	0.0930
	Bra017916	*BrSWEET1b*	0.0400	0.4000	0.1000
AT3G14770	Bra021577	*BrSWEET2a*	0.0700	0.3700	0.1892
	Bra027314	*BrSWEET2b*	0.0500	0.2700	0.1852
AT5G53190	Bra022636	*BrSWEET3a*	0.0900	0.3600	0.2500
	Bra003075	*BrSWEET3b*	0.1200	0.3700	0.3243
AT3G28007	Bra025317	*BrSWEET4a*	0.0500	0.3300	0.1515
	Bra033034	*BrSWEET4b*	0.0600	0.3900	0.1538
AT5G62850	Bra035879	*BrSWEET5a*	0.0600	0.3800	0.1579
	Bra029245	*BrSWEET5b*	0.0800	0.3700	0.2162
AT4G10850	Bra035270	*BrSWEET7a*	0.1200	0.5800	0.2069
	Bra000725	*BrSWEET7b*	0.1500	0.5300	0.2830
AT5G40260	Bra025595	*BrSWEET8*	0.2100	1.2400	0.1694
AT2G39060	Bra000116	*BrSWEET9*	0.0600	0.2800	0.2143
AT5G50790	Bra022761	*BrSWEET10*	0.1000	0.5100	0.1961
AT3G48740	Bra018039	*BrSWEET11a*	0.0500	0.2800	0.1786
	Bra019560	*BrSWEET11b*	0.0500	0.2900	0.1724
AT5G23660	Bra009700	*BrSWEET12a*	0.0600	0.3400	0.1765
	Bra026487	*BrSWEET12b*	0.0400	0.2200	0.1818
AT4G25010	Bra013854	*BrSWEET14a*	0.0700	0.3300	0.2121
	Bra010477	*BrSWEET14b*	0.0700	0.2900	0.2414
AT5G13170	Bra023394	*BrSWEET15a*	0.1100	0.3700	0.2973
	Bra006185	*BrSWEET15b*	0.1100	0.3200	0.3438
	Bra008850	*BrSWEET15c*	0.0900	0.3100	0.2903
AT3G16690	Bra001638	*BrSWEET16a*	0.1600	0.4600	0.3478
	Bra021190	*BrSWEET16b*	0.0800	0.3500	0.2286
AT4G15920	Bra038060	*BrSWEET17b*	0.0600	0.3000	0.2000

### Chromosomal localization of the MtN3/saliva/SWEET genes

**Fig. 1:**
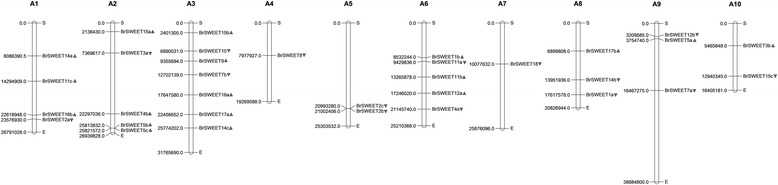
Locations of the MtN3/Saliva/SWEETs genes on the chromosomes of *B. rapa.* The black triangle denotes the transcriptional direction

By chromosomal localization analysis, the 34 MtN3/saliva/SWEETs genes were determined to be distributed among ten chromosomes. By contrast, *BrSWEET13* was distributed on a scaffold. In particular, 7 genes were distributed on the 3rd chromosome; 5 genes on chromosomes 2 and 6; 4 genes on chromosomes 1; and 3 genes on chromosomes 8 and 9; 2 genes were distributed on the fifth and tenth chromosome, while the fourth and seventh chromosome only had one MtN3/saliva/SWEET gene. The MtN3/saliva/SWEETs family genes were scattered across genome, not concentrated on a few chromosomes (Fig. 1).

### Phylogenetic tree analysis of the MtN3/saliva/SWEET in *B. rapa*, *A. thaliana* and *O. sativa*

**Fig. 2:**
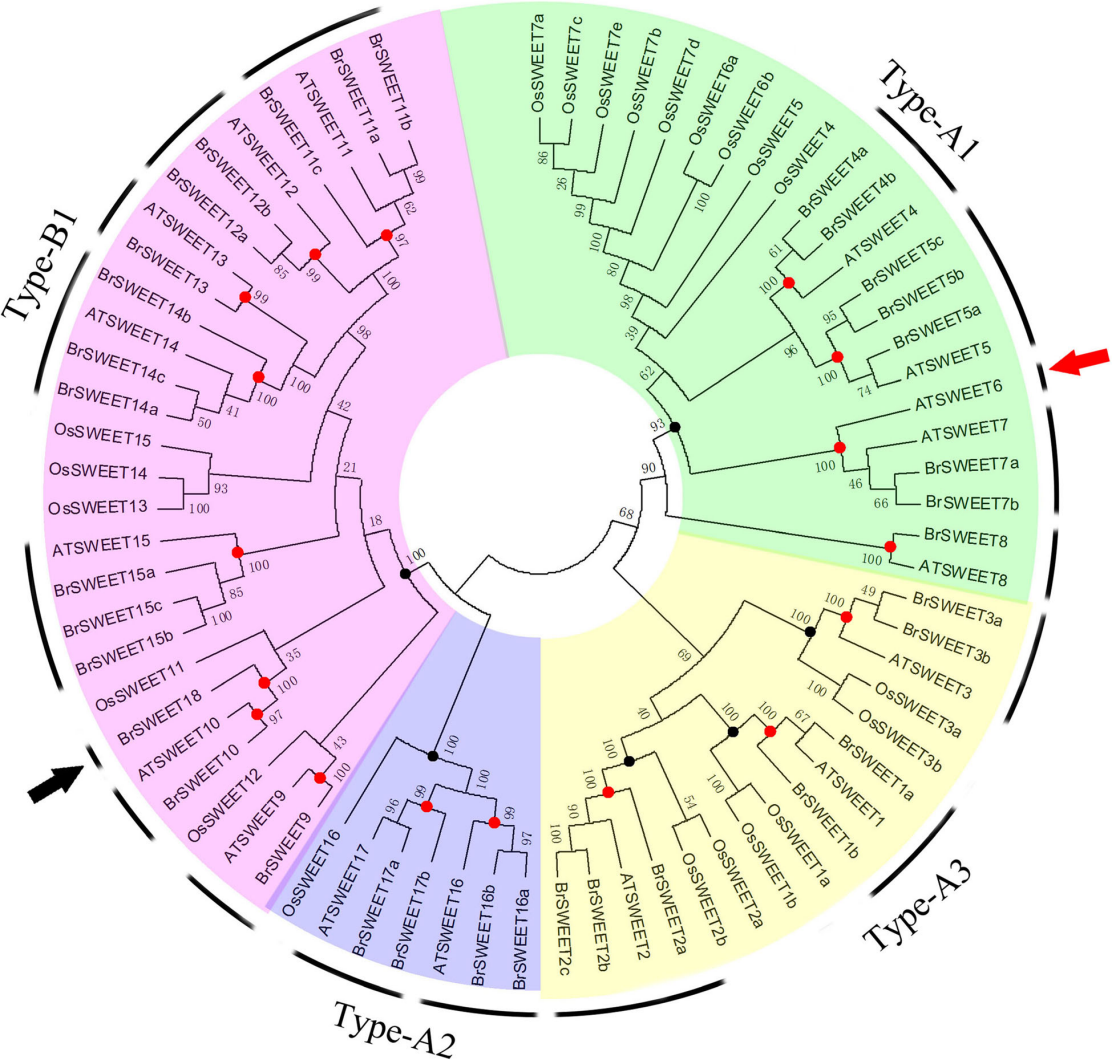
Phylogenetic tree of *B. rapa, A. thaliana* and *O. sativa* MtN3/Saliva/SWEETs genes. The numbers on the branches indicate the bootstrap percentage values calculated from 1000 replicates. The genes in the green, blue, yellow, and purple clades are termed Type-A1, Type-A2, Type-A3, and Type-B1, respectively. The nodes that represent the most recent common ancestral genes before the separation of *B. rapa* and *Arabidopsis* are represented by red circles (bootstrap support> 70%) and black circles mean the possible ancestral genes of *B. rapa, A. thaliana* and *O. sativa* (bootstrap support> 70%). The clades that contain only *B. rapa* and *Arabidopsis* MtN3/Saliva/SWEETs genes are indicated by black and red arrows, respectively

The SWEET genes of *A. thaliana*, *O. sativa*, and *Lycopersicum esculentum* have been classified into four classes, namely, Type-A1, Type-A2, Type-A3, and Type-B1 [33]. As we known, the genome of *B. rapa* has undergone a whole-genome triplication (WGT), therefore, to investigate the retention and loss of the MtN3/saliva/SWEET family genes through evolution, we constructed a phylogenetic tree of all *B. rapa*, *A. thaliana* and *O. sativa* MtN3/saliva/SWEET genes (Fig. 2). Given the existing classification method of *Arabidopsis* MtN3/saliva/SWEET genes, the phylogenetic tree were also classified into four classes. In this study, the MtN3/saliva/SWEETs genes in the green, blue, yellow, and purple clades were termed as Type-A1, Type-A2, Type-A3, and Type-B1, respectively, and the clades contained 8, 4, 7, and 15 *Brassica* MtN3/saliva/SWEETs family members, respectively. Twelve pairs of protein sequences (one pair means they have the same homologous gene in *Arabidopsis*), for example, BrSWEET1a*/BrSWEET1b*, exhibited high similarity to each other and were located in closely related branches. These properties indicated the functional redundancy of such sequences. Furthermore, *BrSWEET7, BrSWEET9*, *BrSWEET8*, and *BrSWEET10* showed a relatively low homology with the other MtN3/saliva/SWEET family genes. One branch only contained the *B. rapa* MtN3/saliva/SWEET genes (Fig. 2, black arrows) and one branch only contained the *Arabidopsis* MtN3/saliva/SWEET genes (Fig. 2, red arrows). To analyze the number of nodes in the phylogenetic tree, we found 17 nodes with high confidence values (> 70%). This result indicated the existence of at least 16 supposed ancestor genes of *A. thaliana* and *B. rapa* (red dots); six nodes with the same standard mean the possible ancestor genes of *A. thaliana*, *B. rapa* and *O. sativa* (Fig. 2, black dots).

The gains and losses, as well as the copy number changes, during the evolution of the *Arabidopsis* and *B. rapa* MtN3/saliva/SWEET genes in different clades were analyzed in this study (Additional file 3: Figure S1). The numbers of *Arabidopsis* and *B. rapa* MtN3/saliva/SWEET genes in clade type A1 were 5 and 8, respectively, moreover, the number of putative common ancestor genes was 4; the number of genes gained were 4 and 1, respectively, and the number of genes lost were 0 and 0, respectively. Clade type A2 was the branch with the lowest numbers of MtN3/saliva/SWEET genes at 2 and 4 for *Arabidopsis* and *B. rapa*. These genes had 2 ancestor genes. The *Arabidopsis* MtN3/saliva/SWEET genes did not show gains and losses, whereas *B. rapa* gained 2 MtN3/saliva/SWEET genes. *Arabidopsis* and *B. rapa* attained 3 and 7 MtN3/saliva/SWEET genes in clades type A3, and these genes had 3 putative common ancestor genes and gained 4 and 0 genes, respectively, and lost 0 and 0 genes, respectively. Clade type B1 was the largest group with 15 *B. rapa* MtN3/saliva/SWEET genes and 7 *Arabidopsis* MtN3/saliva/SWEET genes. They have 8 supposed ancestor genes, with gaining 7 genes gained in *B. rapa* and 1 gene lost in *A. thaliana.*

### Ka and K_S_ calculation of orthologous SWEET genes between *B. rapa* and *A. thaliana*

The ratio of the nonsynonymous substitution rate to the synonymous substitution rate is a significant indicator of the selection pressure acting on genes being assessed. Ka/Ks > 1, ≈1, and < 1 indicate positive Darwinian selection, neutral selection, and purification selection, respectively [34]. We calculated the Ka/Ks ratios of the orthologous SWEET genes between *B. rapa* and *A. thaliana*. These ratios were far less than 1, which imply that the orthologous genes suffered strong purifying selection for retention (Table 2).

**Fig. 3:**
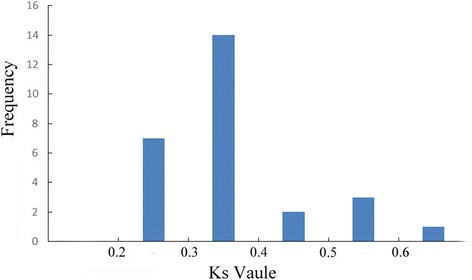
The *K*_s_ values distribution of MtN3/Saliva/SWEETs genes in the genome of *B. rapa* and *A. thaliana*. The vertical axis indicates the frequency of paired sequences, whereas the horizontal axis denotes the *K*_s_ values with an interval of 0.1

The *K*_s_ values obtained from comparisons of the sets of putative orthologs were used to calculate the divergence time between *B. rapa* and *A. thaliana* (Table 2). The *K*_s_ values Concentrate in areas 0.3 to 0.4 (Fig. 3). The calculation of divergence time was based on the neutral substitution rate of 1.5 × 10^− 8^ substitutions per site per year for *Chs* [25]. Our results showed the MtN3/saliva/SWEET gene family of *B. rapa* diverged from *A. thaliana* at around 10 MYA to 13 MYA (Million Years Ago).

### Structural analysis of MtN3/saliva/SWEET genes

**Fig. 4:**
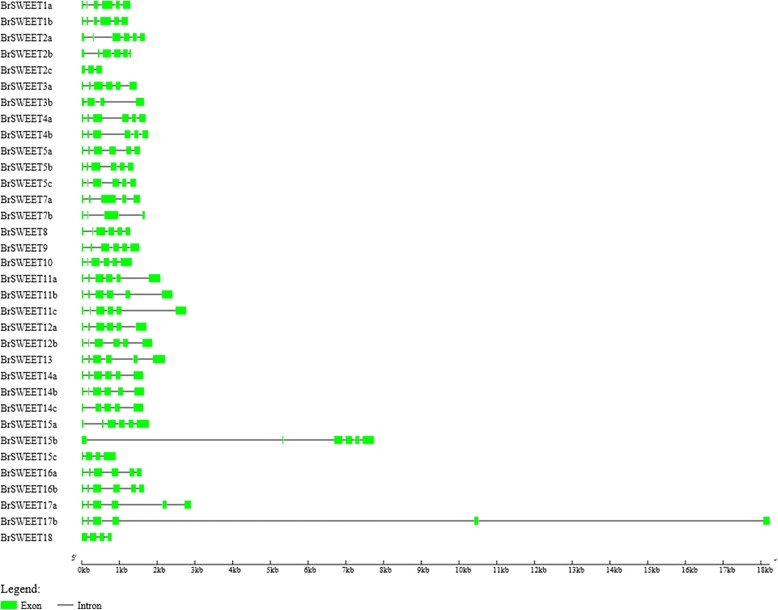
Evolutionary and exon–intron analyses of the MtN3/Saliva/SWEETs genes of *B. rapa.* Exons and introns are represented by boxes and lines, respectively

The results of the intron–exon location analysis showed that the number and distribution of the introns and exons of MtN3/saliva/SWEET genes were highly conserved (Fig. 4). A total of 26 members of the MtN3/saliva/SWEET family harbored six exons. *BrSWEET2c* has three exons. *BrSWEET3b, BrSWEET7b, BrSWEET15c* and *BrSWEET18* contained four exons, *BrSWEET7a BrSWEET14c* and *BrSWEET 15b* contained five exons. *BrSWEET17b* comprised a special structure with six exons, three short introns and two long introns.

The amino acid sequences of the 29 MtN3/saliva/SWEET family genes were aligned with ClustalW. The results reveal that the intramembranous areas of the MtN3/saliva/SWEET domain were highly conserved. Furthermore, the transmembrane regions were relatively conserved, and the two highly conserved regions may have each contained a serine phosphorylation site (Additional file 4: Figure S2). Hence, the intramembranous areas of the MtN3/saliva/SWEET family protein may correspond to important functional or regulatory regions of the protein structure. The regulatory methods may be reversible phosphorylation/dephosphorylation pathways controlled by a number of protein kinases/phosphatases. This information can be an important entry point for the functional analysis of *Arabidopsis* MtN3/saliva/SWEET family genes.

### Promoter sequences analysis of MtN3/saliva/SWEET genes

To further understand the transcriptional regulation and potential function of the MtN3/saliva/SWEET genes, we analyzed the *cis*-elements in the promoter sequences (Additional file 5: Table S3). There are more than half *cis*-acting elements involved in light response elements in the development associated elements, these underlined elements are all light response elements. Most of MtN3/saliva/SWEET genes contained one or more *cis*-elements involved in photoresponse, these are ACE, Box4, BoxI, G-box, GAG-motif, GT1-motif, MRE, Sp1, TCCC-motif, ATCT-motif, TCT-motif, GAG-motif, and CATT-motif. Therefore, we hypothesized that the MtN3/saliva/SWEET family genes are involved in plant photosynthesis. Some MtN3/saliva/SWEET genes contained the *cis*-elements involved in hormonal response. A total of 14 MtN3/saliva/SWEET genes contained the ABRE element involved in ABA response. Then, 15 MtN3/saliva/SWEET genes contained the GARE-motif involved in gibberellin response. Meanwhile, 25 genes harbored the TGACG-motif and CGTCA-motif involved in the MeJA response. A total of 13 genes contained the TGA-element involved in auxin response. In contrast, 22 genes contained the TCA-element involved in the salicylic acid response. Otherwise, the scope of the stress-response-related *cis*-acting elements was relatively broad and includeed high-temperature stress, low-temperature stress, drought-induced stress, anaerobic induction, and other related components. A total of 26 genes contained HSE involved in the high-temperature stress response, whereas 17 genes contained LTR involved in the low-temperature stress response. Statistical analyses suggested that more than half of the *cis*-elements were light-responsive elements. About one-third were related to stress, and the rest were associated with growth and development. Hence, the MtN3/saliva/SWEET genes may be regulated by light signals. The *cis*-elements present in the promoter regions of the MtN3/saliva/SWEET genes revealed their essential role in growth and development, as well as in mediating responses to abiotic stresses.

### Analysis of the secondary structure of the MtN3/saliva/SWEET proteins in *B. rapa*

NPSA-MLRC (https://npsa-prabi.ibcp.fr/cgi-bin/npsa_automat.pl?page=/NPSA/npsa_mlrc.html) [35] was employed to predict the secondary structure of the MtN3/saliva/SWEET proteins. The main secondary structures of the MtN3/saliva/SWEET proteins were the α-helix, irregular curl, and extended strand. Most of MtN3/saliva/SWEET proteins exhibited some similarities in the proportion of constituent secondary structural elements. That is, approximately 15%–40% were α-helices, approximately 40%–50% were irregular curls, and approximately 20%–40% were extended strands (Additional file 6: Table S4).

### Transmembrane regions and hydrophobicity analysis of the MtN3/saliva/SWEET proteins

**Fig. 5:**
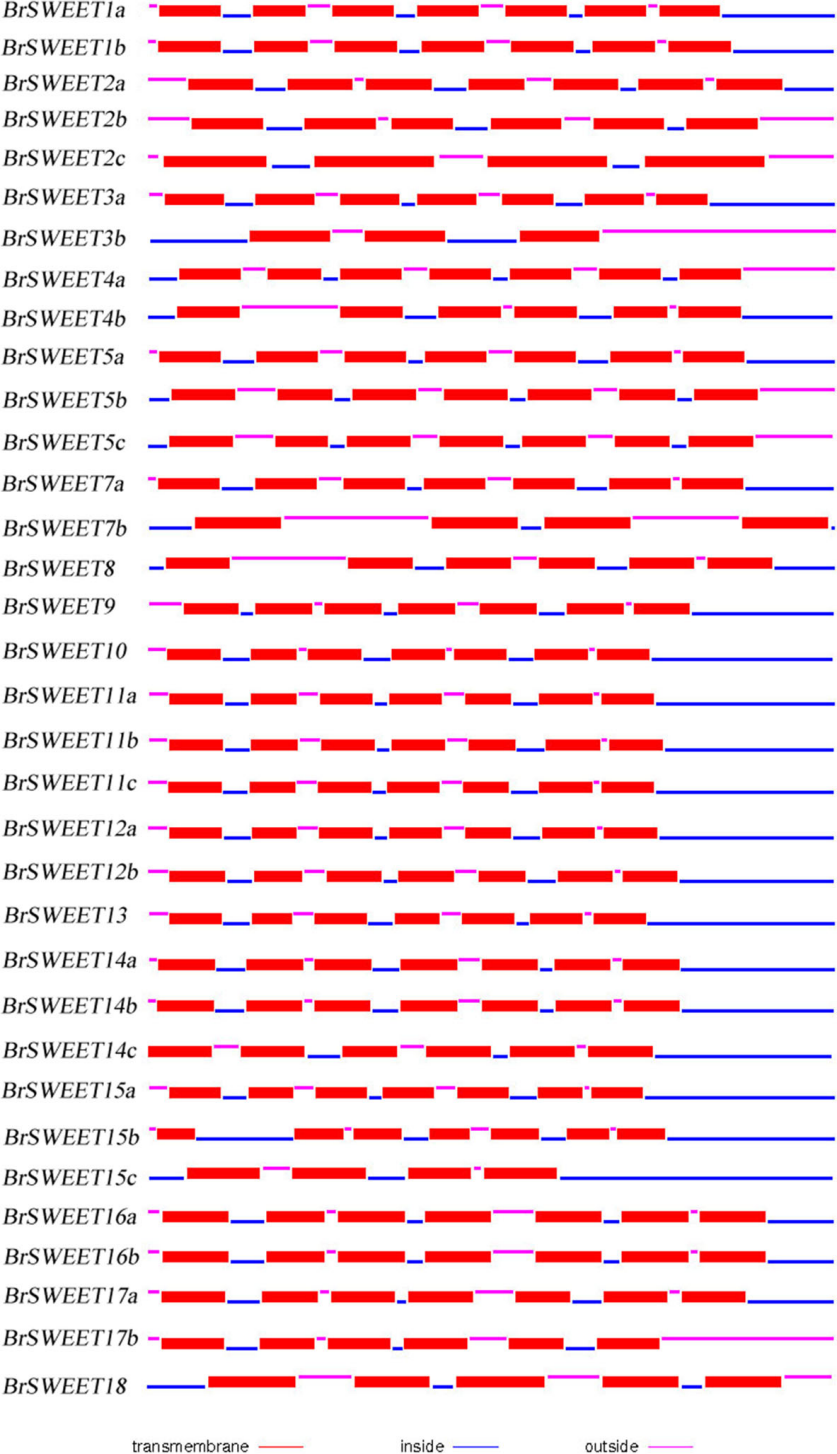
Protein structure of MtN3/Saliva/SWEETs in *B. rapa.* The blue lines signify the intracellular region. The thick red line denotes the transmembrane region. The red lines indicate the extracellular region

Using TMHMM software to predict the transmembrane regions of the 34 MtN3/saliva/SWEET proteins, we found that 24 members of the encoded protein harbored seven transmembrane regions. Five members contained six transmembrane regions, one harbored five transmembrane regions, and two members contained four transmembrane regions. These results imply that the MtN3/saliva/SWEET family proteins likely localize in the membrane or membrane receptors (Fig. 5).

The hydrophobicity analysis revealed that all the MtN3/saliva/SWEET proteins were hydrophilic proteins (Additional file 7: Table S5).

### Signal peptide analysis and subcellular localization analysis of the MtN3/saliva/SWEET family proteins

Signal peptide analyses of the 34 MtN3/saliva/SWEET proteins were performed on the basis of the neural network model of the SignalP 4.1 Server. A total of 34 MtN3/saliva/SWEET proteins did not contain a signal peptide sequence.

To understand the location of the 34 MtN3/saliva/SWEET proteins in the cell, we performed subcellular localization. *BrSWEET2a*, *BrSWEET16a*, *BrSWEET16b*, *BrSWEET17a*, and *BrSWEET17b* are likely positioned at the vacuolar membrane, whereas *BrSWEET1a*, *BrSWEET1b, BrSWEET3a* and *BrSWEET3b* may be located in the chloroplast. The rest are more likely located in the plasma membrane except *BrSWEET2c,* which may be located in extracellular domain (Additional file 8: Table S6).

### Conserved short amino-acid sequence analysis of the MtN3/saliva/SWEET proteins in *B. rapa*

**Fig. 6:**
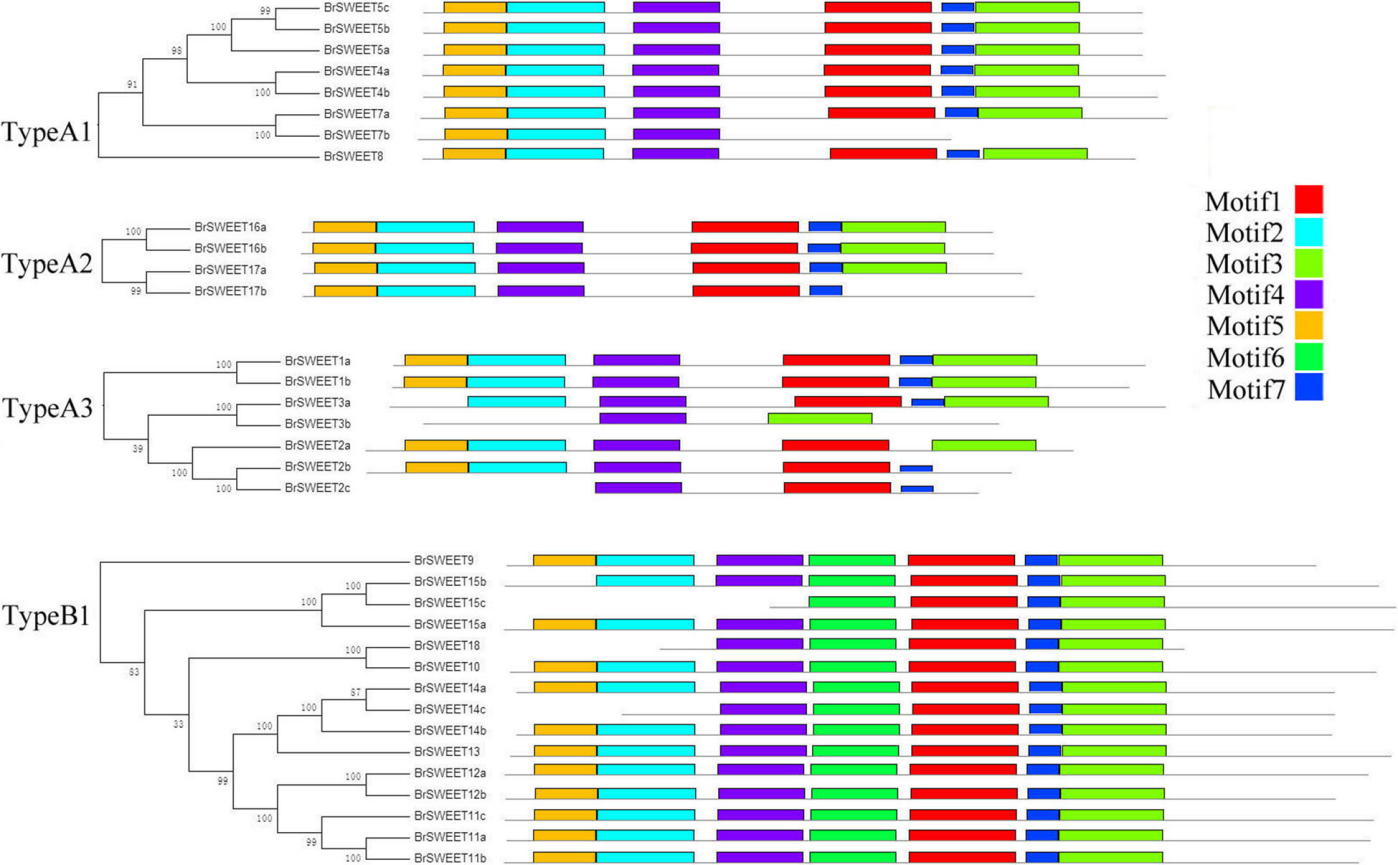
MEME analysis of the conserved motifs of SWEET genes in *B. rapa*

MEME analysis showed seven conserved motifs of MtN3/saliva/SWEET existing in *B. rapa* (Fig. 6 and Additional file 9: Figure S3)*.* Most members in the Clade type A1, typeA2 and typeA3 have 6 motifs except motif 6, while most genes in Clade type B1 have 7 motifs. BrSWEET2c only has motif 1, 4 and motif 7; BrSWEET3b only has motif 3 and motif 4; BrSWEET7b only has motif 2, 4 and motif 5. These genes only have two or three motif, because they just have one domain. The result show that TypeA and TypeB have its own conservative motif and our classification is reasonable.

### Expression characterization of the MtN3/saliva/SWEET genes

Tissue-specific and developmental stage-related expression data provides us with clues about the function of the MtN3/saliva/SWEET genes in different tissues and floral organs. Thus, we obtained an initial picture of the functions of the MtN3/saliva/SWEET genes during vegetative and reproductive development. To achieve this goal, we analyzed the transcript levels of the genes in the roots, stems, leaves, flowers, immature siliques, sepals, petals, stamens, pistils, nectaries, and small and large buds by qRT-PCR.

**Fig. 7:**
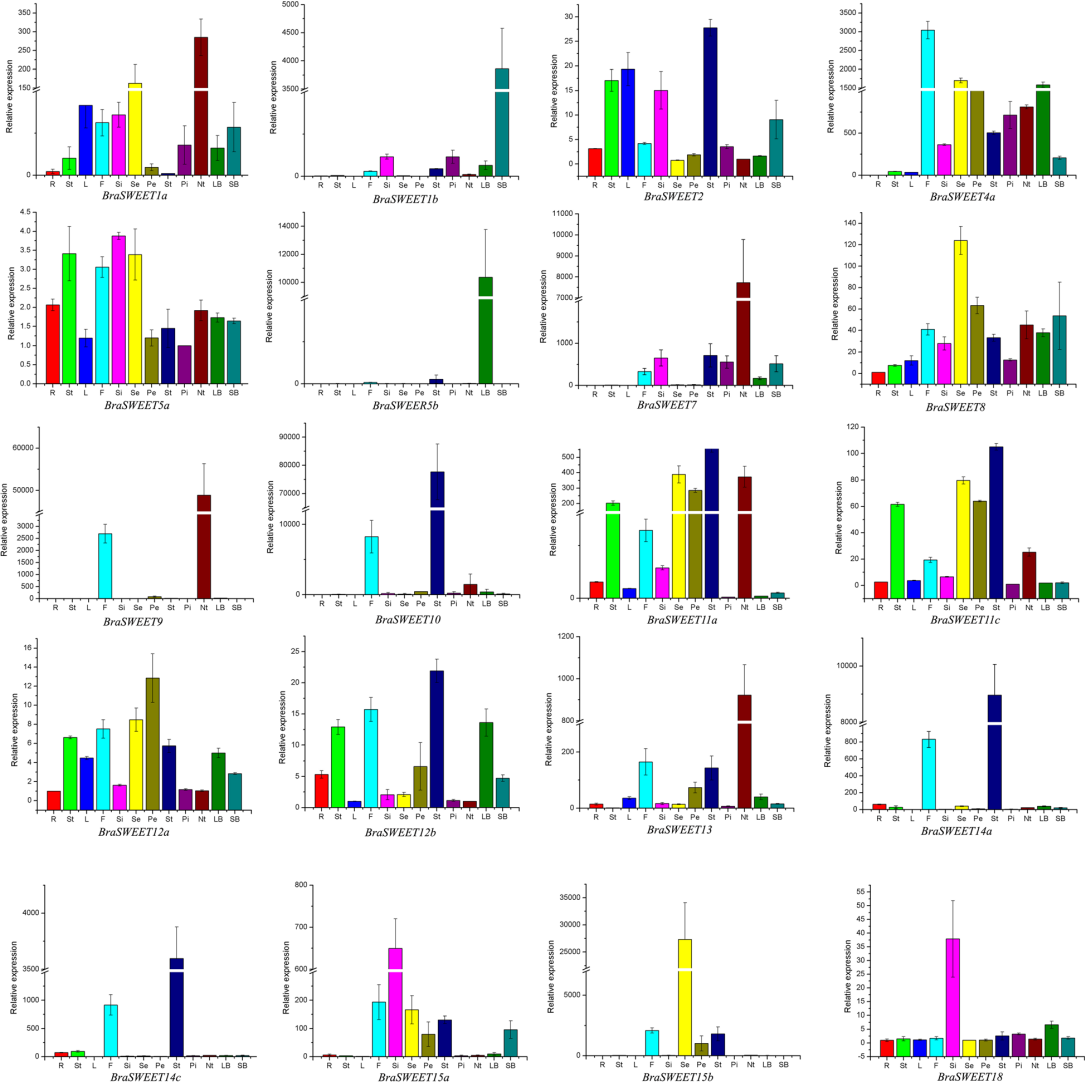
Relative expression of MtN3/Saliva/SWEETs genes analyzed by qRT-PCR in different tissues and floral organs. R: root; St: stem; L: leaves; F: flower; Si: silique; Se: sepal; Pe: petal; St: stamen; Pi: pistil; Nt: nectary; LB: large bud; and SB: small bud

From the qRT-PCR results of samples from different tissues (Fig. 7), we found that the expression patterns of most of the MtN3/saliva/SWEET genes were tissue specific, except for the amplified products from primers named 021577, 016421, 026487,025595, 009700, and 035879. Most of the expression differences were more than 100 times different. The transcripts of *BrSWEET11c* and *BrSWEET11a* were specifically expressed in the floral axis. Thus, the genes may be related to floral axis development. Meanwhile, the expression patterns of *BrSWEET13*, *BrSWEET14a*, *BrSWEET14c*, *BrSWEET10*, *BrSWEET7*, *BrSWEET9*, *BrSWEET4a*, *BrSWEET5c*, and *BrSWEET15b* were relatively specific in the flower. Thus, these genes may serve roles in the reproductive developmental process of the Chinese cabbage. *BrSWEET15a*, *BrSWEET7*, *BrSWEET18*, and *BrSWEET1b* were specifically expressed in siliques and may be involved in reproductive development. In summary, the MtN3/saliva/SWEETs gene family may be involved in the reproductive developmental process of Chinese cabbage.

qRT-PCR of the MtN3/saliva/SWEET genes from the different floral organs showed that the gene expression levels of the different MtN3/saliva/SWEET genes evidently differed among the different flower organs (Fig. 7). The transcripts of *BrSWEET11c* and *BrSWEET11a* were expressed at a significantly higher level in sepals, petals, stamens, and nectaries than in the other parts of the flower. By contrast, *BrSWEET15a* was relatively highly expressed in the sepals, petals, stamens, and small flower buds. Some genes were specifically expressed in different floral organs. *BrSWEET13*, *BrSWEET7*, *BrSWEET1a*, and *BrSWEET9* were specifically expressed in the nectary. *BrSWEET14a*, *BrSWEET14c*, and *BrSWEET10* were specifically expressed in the stamen. *BrSWEET15b* was specifically expressed in the sepals. *BrSWEET5c* and *BrSWEET1b* were specifically expressed in the large and small flower buds. *BrSWEET*4a and *BrSWEET8* exhibited relatively high expression levels in various floral organs. By contrast, *BrSWEET12a*, *BrSWEET5a*, *BrSWEET12b*, and *BrSWEET18* revealed almost no expression in various flower organs.

### Subcellular localization analysis and overexpression analysis of *BcNS* in *B. rapa*

**Fig. 8:**
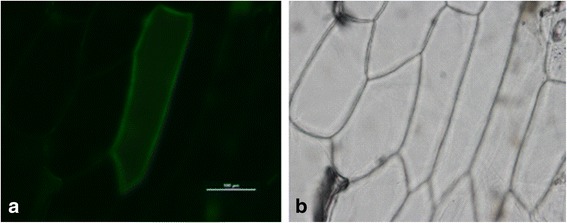
Subcellular localization analysis of *BcNS* in onion epidermal cells. **a** Yellow fluorescent protein signal visible in the onion epidermal cells with pB7YWG2.0-*BcNS*-YFP **b** Bright field of the corresponding onion epidermal cell. Scale bar = 100 μm

To explore the subcellular localization of BcNS, we constructed a pB7YWG2.0-*BcNS*-YFP overexpression vector and transiently expressed the gene in onion epidermal cells via gene-gun bombardment. The transient expression of *BcNS* in onion epidermal cells showed that the protein was located on the cell membrane (Fig. 8). This result is consistent with the predicted results (Additional file 8: Table S6; Additional file 10: Figure S4).

**Fig. 9:**
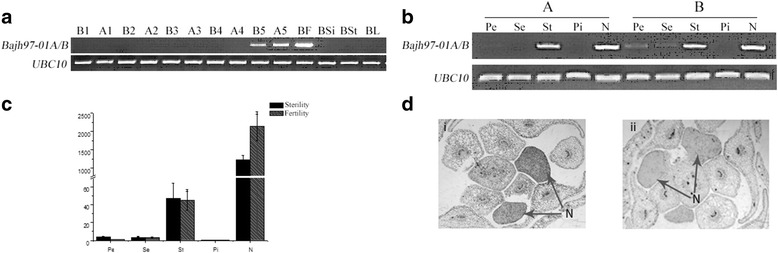
The expression pattern of *BcNS* in *B. rapa*. **a** The RT-PCR expression results of *BcNS* in a recessive sterile A/B line ‘*Bajh97-01A/B*’; **b** The RT-PCR expression results of *BcNS* in floral organs of a recessive sterile A/B line ‘Bajh97-01A/B’; **c** The relative expressional levels of *BcNS* in different organs of the flower; **d** Analysis of the expression pattern of *BcNS* using in situ hybridization, i: hybridization results with the antisense probe; ii, hybridization result with the sense probe. 1, 2, 3, 4, and 5 indicate stage I (< 1 mm), stage II (1.2 ~ 1.6 mm), stage III (1.8 ~ 2.2 mm), stage IV (2.4 ~ 2.8 mm), and stage V (3.0 ~ 3.4 mm) flower buds, A: fertile line, B: sterile line, F: open flowers, Si: germinal siliques, St: stems, L: leaves, Pe: petal, Se: sepal, St: stamen, Pi: pistil, and N: nectary

Results of semi-quantitative PCR showed that *BcNS* was expressed in V buds and opened flowers and there was not significant difference between sterile plants and fertile plants (Fig. 9a). To further understand the expression pattern of the *BcNs*, we detected in expression in different flower organs. The results revealed that *BcNS* was specifically expressed in the stamen and nectary (Fig. 9b). These results are consistent with quantitative reverse-transcription PCR (Fig. 9c). Meanwhile, result of in situ hybridization indicated that *BcNS* was expressed in nectary tissue (Fig. 9d).

We also constructed the antisense expression vector *pCAMIA1301-BcNS* and transformed this vector into *B. rapa* by *Agrobacterium*-mediated transformation to study the biological function of *BcNS* (Additional file 11: Figure S5). In this study, the transgenic plants were detected by PCR, X-Gluc, and fluorescence quantitative PCR. These methods provided reliable materials for functional verification (Additional file 12: Figure S6). We obtained 45 transgenic germ lines and 22 positive lines after PCR detection. The positive rate was approximately 50%. We detected the calluses and leaves of the transgenic plants with the X-Gluc histochemical assay. The calluses were stained blue, and the leaves were stained with difficulty. Hence, the antisense *BcNS* gene fragment was integrated into the genomic DNA in *B. rapa.* The transgenic plants detected by PCR and GUS staining were analyzed by qRT-PCR. Expression in the transgenic plants decreased relative to that in the negative control.

**Fig. 10:**
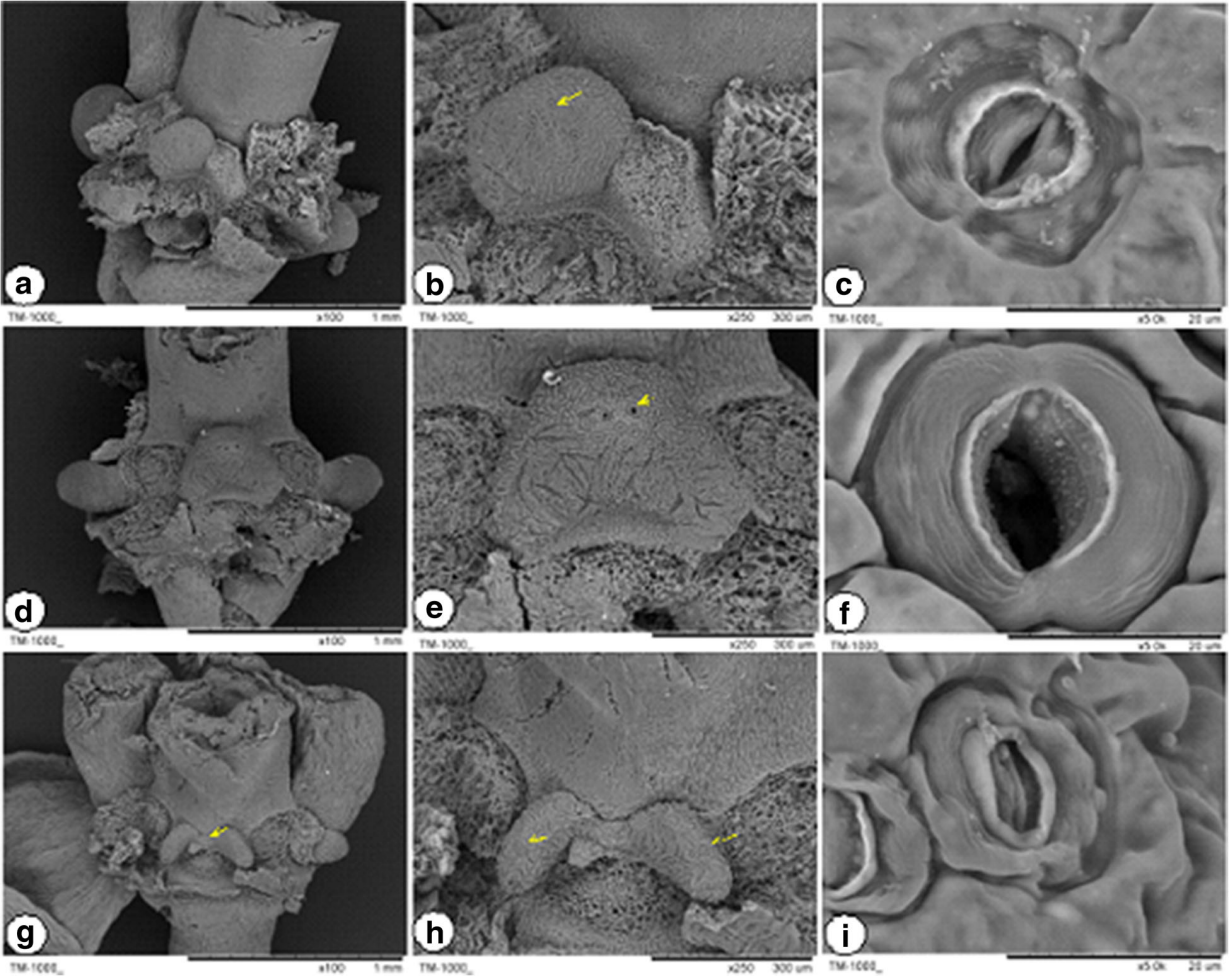
Scanning electron microscopy observation of the nectaries in the transgenic plants and their control in *B. rapa* ssp. *chinensis* var. *parachinesis*. **a**–**c** are wild types, **d**–**g** are pCAMBIA transgenic plants, **h**–**i** are pCAMBIA*-BcNS* transgenic plants. The arrow in H refers to the forking of the nectary development. The arrows in **b**, **e**, and G refer to the stoma of the nectary. The results indicate that not all of the nectaries were constantly open (**f**); some were closed (**i**) or half-closed (**c**)

We observed the flower organ development of the transgenic plants, controls, and wild types. Unlike in the wild type, the petals, stamens, and pistils in the transgenic plants and the controls were normal. Nevertheless, we found splitting phenomena at the base of the lateral nectarines (Fig. 10 and Additional file 13: Figure S7), and the proportion of affected nectaries exceeded 70% (Table 3).

**Table 3:** The result of abnormal nectary development between the transformants and their controls

Materials	No. of flower with normal nectary	No. of flower with abnormal nectary	Ratio of flower with abnormal nectary(%)
WT	34.67 ± 4.16	6.33 ± 0.58	15.50% ± 1.19%
CK	35.00 ± 2.94	7.00 ± 0.81	16.70% ± 1.76%
Transformants of A-12	12.33 ± 2.52	31.67 ± 4.73	71.80% ± 6.23%
Transformants of A-13	13.33 ± 3.21	35.00 ± 2.65	72.49% ± 5.91%

Three plantets and about 15 flowers per plantet have been employed to investigate the abnormal nectary development, respectively

Thereinto, the transformant of A-20 damped off before flowering, and did not get the observation data

## Discussion

MtN is *M. truncatula* Nodulin and was first reported in 1996 [3]. A new sugar transporter family was found by sugar optical sensors and named the SWEET family in 2010 [7]. Afterward, SWEETs and the gene family MtN3/saliva were reported to be the same [6]. Currently, relatively large-scale studies are available on MtN3/saliva/SWEET involved in plant pathogen invasion and reproductive development. The MtN3/saliva/SWEET family proteins are involved in anther dehiscence, pollen wall formation, and other gametophyte developmental processes in *Arabidopsis*, petunia, and rice [9–11].

The genus *Brassica* is known for its important economic crops, such as Chinese cabbage, cabbage, broccoli, cauliflower, rutabaga, turnip and some seeds used in the production of canola oil and condiment mustard. To date, there are a number of gene families that have been identified in *Brassica* [22, 36, 37]. In this study, we successfully identified 34 MtN3/saliva/SWEET genes based on the *Arabidopsis* Genome Database TAIR and *Brassica* database. There genes were termed as Type-A1, Type-A2, Type-A3, and Type-B1in the same way as rice and tomato [38, 39]; the clades respectively contained 8, 4, 7, and 15 members in *B. rapa*, respectively. Physiological and biochemical property analyses revealed that the total lengths of the coding regions of the genes were 350–1000 bp, the molecular weights were 14–35 kDa, and the pIs were 7.59–9.62. The 34 genes were distributed on various chromosomes. After analyzing the *cis*-acting elements of the promoters of these genes, we found that most were related to optical response. Therefore, we hypothesized that the MtN3/saliva/SWEET are likely involved in plant photosynthesis. Gene Ontology analysis of the turnip nectary ESTs, floral organ ESTs, root ESTs, and 12,448 tomato nectary ESTs showed that the photosynthesis-related lateral nectary ESTs (MLN ESTs) were even richer than the median nectary ESTs (MMN ESTs) [18]. Although numerous floral organ ESTs and tomato nectary ESTs exist, other biological processes are more prominent than the photosynthetic process. Most turnip nectary-specific genes are involved in photosynthesis. Therefore, the results of the *cis*-acting element analysis are highly meaningful in the study of *Brassica* nectar carbohydrates.

After predicting the transmembrane regions of the MtN3/saliva/SWEET proteins, we found that all the members comprised transmembrane regions. That is, 24 members contained seven transmembrane regions, and the extracellular regions of these proteins were generally very short. The intramembrane areas were highly conserved regions, whereas the transmembrane regions were relatively conserved. The two highly conserved regions may harbor amino acid phosphorylation sites. These results indicate that the intramembrane areas are important functional areas of the protein. Furthermore, the regulatory method may be reversible phosphorylation/dephosphorylation pathways controlled by a number of protein kinases/phosphatases. These findings can be an important entry point for the functional analysis of the MtN3/saliva/SWEET family genes.

Evolutionary analysis showed that some genes with known physiological functions exist in a relatively distant branch on the evolutionary tree. These genes possibly play a separate role in the developmental process. Our results showed MtN3/saliva/SWEET gene family of *B. rapa* diverged from *A. thaliana* at around 10 MYA to 13 MYA, which is a little later than a previous study, which have confirmed the genome of *B. rapa* diverged from *A. thaliana* lineage at least 13 ~ 17 MYA [40–42]. In the previous study, the result was based on the whole genome, while our results was gained from the MtN3/saliva/SWEET gene family which only have 34 members. As we known, *A. thaliana* and Brassicaceae taxa have undergone three times (γ, β and α) of the whole genome duplication or triplication (WGD or WGT) [43]. After the α duplication, *Arabidopsis* lineage separated from its *Brassica* ancestor approximately 43.2 MYA [40]. In the long river of species evolution, the difference of the divergence time is acceptable. These results hint that the MtN3/saliva/SWEET gene family is a good molecular reference candidate to be utilized in the study of plant evolution analysis. Our results further confirmed the hypothesis that *Brassica* genomes have undergone another whole genome triplication (WGT) after speciation from *A. thaliana.* The existence of gene duplications in Chinese cabbage and *Arabidopsis* that occurred during 3R also demonstrated their conservation during the long-term evolution [40, 44]. Meanwhile, another obvious distribution peak of the relative *Ks* appeared 1.24, which was responsible for the 3R event before the speciation between *Brassica* and *Arabidopsis* genomes. In addition, MtN3/saliva/SWEET family genes involve different physiological functions in plants, including reproductive development, senescence, abiotic stress response, and host–pathogen interactions. At least seven members (*AtSWEET2*, *AtSWEET4*, *AtSWEET7*, *AtSWEET8*, *AtSWEET10*, *AtSWEET12*, and *AtSWEET15*) can be induced by the genus *Pseudomonas* DC3000 in *Arabidopsis* [45]. Moreover, *AtSWEET4*, *AtSWEET15*, *AtSWEET17*, *AtSWEET11*, and *AtSWEET12* can be induced by other elicitor types [45, 46]. *RPG1* (*AtSWEET8*) is involved in the pollen wall development process [11]. *SAG29* (*AtSWEET15*) is mainly expressed through the abscisic acid pathway depending on the osmotic pressure in plant senescent tissues [12]. Silencing *Xa13/Os8N3/OsSWEET11* can significantly increase the resistance to PXO99 (Xa13 nonaffinity strains) in rice; thus, Xa13 is essential for PXO99 in rice [7]. These findings provide information for future biological function research on other MtN3/saliva/SWEETs family genes.

The gene expression levels of the different MtN3/saliva/SWEET genes evidently differed among different tissues and flower organs. Many genes were specifically expressed in different floral organs. The MtN3/saliva/SWEET gene family was found to be involved in reproductive development in Chinese cabbage. *RPG1 (RUPTURED POLLEN GRAIN1/Sweet8)* encodes a membrane protein in *Arabidopsis*; this protein is essential for small exine formation [11]. One study showed that *RPG1* affects cell integrity, whereas the fertility of mutations are partially repaired during late reproductive development. The authors speculated that the gene was partially supplemented by *RPG2*. These results indicated that *RPG1* and *RPG2* interact in pollen wall sediment formation and consequently affect pollen wall formation [47]. In *A. thaliana* and rice, expression pattern analysis revealed additional genes from the MtN3/saliva/SWEET family that were involved in reproductive development. At least six SWEET genes were highly expressed in flowers at different developmental stages in rice. A number of genes were highly expressed in *Arabidopsis* inflorescences. *LIM7* was found to be induced in the early meiotic prophase of lily microsporocyte in lily [48]. *Lestd1* is specifically expressed in mature pollen grains in tomato [49]. *GO255182* plays an important role in the interaction between the pollen and stigma in *Senecio squalidus* [50]. The expression of *TOBC023B06* is significantly high in tobacco [51]. These data demonstrated that the MtN3/saliva/SWEET gene family is closely related to reproductive developmental processes.

Bacterial and fungal pathogens were vaccinated to verify the change in the mRNA contents of the MtN3/saliva/SWEET gene family members in *Arabidopsis*. The expression of *AtSWEET4*, *AtSWEET5*, *AtSWEET7*, *AtSWEET8*, *AtSWEET10*, *AtSWEET12*, and *AtSWEET15* was significantly increased in the leaves [45]. In pepper, the TAL effector AvrBs3, which is secreted by the bacterium *Xanthomonas*, can induce the expression of *UPA16* [52]. In summary, the MtN3/saliva/SWEET gene family may play a key role in the plant reproductive developmental process. That is, the gene family may improve the immune system and protect against stress responses. The abovementioned studies provide important clues for clarifying the biological function of the gene family members.

Numerous genes are acquired or lost in the evolution process. In Chinese cabbage, 11 pairs of duplicated SWEET genes were retained: *BrSWEET1a/1b*, *BrSWEET2a/2b, BrSWEET3a/3b, BrSWEET4a/4b*, *BrSWEET5a/5b*, *BrSWEET7a/7b, BrSWEET11a/11bc*, *BrSWEET12a/12b*, *BrSWEET14a/14b*, *BrSWEET15a/15bc* and *BrSWEET16a/16b*. Retention and loss originated directly from species needs. The duplication genes generally believed to be retained are those involved in neofunctionalization, subfunctionalization, and nonfunctionalization [53]. In the neofunctionalization model, one copy of the duplicated gene gains a new function by accumulating a favorable mutation, whereas the other copy retains the original function. In the subfunctionalization model, one gene retains some of the functions of the ancestral gene, whereas the other gene retains the original function. In the nonfunctionalization model, one of the copies is silent due to degeneration, whereas the other copies retain the original function. Neofunctionalization and subfunctionalization can lead to the differential spatial and temporal expression of duplication genes [53]. No similarity was noted between the expression patterns of the 7 pairs of SWEET duplication genes in Chinese cabbage. This result may be due to the neofunctionalization or subfunctionalization of the genes.

Protein function is closely related to protein subcellular localization [54]. To determine the location of genes related to nectary development in *B. rapa*, we studied the subcellular localization characteristics of the *BcNS* gene by the YFP-labeling method based on the Chinese cabbage cultivar ‘Chiifu-401.’ The protein was located on the cytoplasmic membrane; hence, the gene may play a role in the stability of the cell membrane or the control of protein transport and other physiological and biochemical processes.

The expression pattern of *BcNS* in different stages of flower development showed that the gene was expressed in fifth buds. There was no significant difference between sterile and fertile lines, which indicates that the gene may not be involved in pollen fertility. To further prove our conjecture, we examined the expression of the gene in different flower organs of ‘*Bcajh97-01A/B*’ and ‘Chiifu-401’. The results showed that the gene is specifically expressed in nectaries and stamens. One MtN3/saliva family gene, *NEC1*, is also expressed in nectaries and stamens, and plays an important role during anther development. We had an assumption that *BcNS* may be related to nectaries and anther development in Chinese cabbage. The antisense genetic method has been used to studying the function of the MtN3/saliva family genes [10, 11, 55]. We cloned an MtN3/saliva gene and named the gene as *BcNS*. This gene is highly expressed in the nectaries and stamen. We then constructed an antisense expression vector *pCAMIA1301-BcNS* and obtained transgenic plants. Morphological observations revealed a splitting phenomenon at the base of the lateral nectarines of the transgenic plants. However, detailed mechanisms of the phenotype remains to be further studied. Nectaries secrete nectar to attract insects for pollination [56]. The nectary is necessary for seeds formation, and the removal of the nectary from the flower of Christmas bells caused an increase in nectar production, but a decrease in the ability to produce seeds [57]. Many studies suggest that nectary development is dependent on hormonal signaling. For example, *PIN6*, an auxin efflux transporter factor, positively regulated nectar production [58]. Jasmonic acid (JA) can promote nectar secretion [59]. Additionally, overexpression of *GA2ox6* (*GA 2-OXIDASE6* (*GA2ox6*, *At1g02400*)) increased nectar sugar in nectaries, suggesting that GA negatively regulates nectar production [60].

Previous studies similarly reported on the nectarine split. *NEC1* had differential expression in petunia with high expression in anther dehiscence cells, nectaries, and upper stamen filaments. *Nec1* possesses an early anther dehiscence forward phenotype [9]. The YABBY transcription factor genes *CRABS CLAW* (*CRC*) plays a role in the early development of nectaries and carpels in *Arabidopsis* [61]. Some genes that regulate CRC expression are also involved; examples include the B-type gene (*APETALA3* and *PISTILLATA*) and the C-type gene (*AGAMOUS* and *SEPALLATA*). The study showed that CRC expression was very high in the early stage of development and throughout the whole secretory period [62]. Therefore, *CRC* plays an important role in the development and secretion of nectar in nectaries. In addition, *bop1* and *bop2* double mutants could not form a normal nectary, but the expression of *CRC* in these mutants is normal [15]. We speculated that the coordinated interaction of the three genes is the basis for the production of a normal nectary. In future research, we intend to focus on analyzing whether *CRC*, *BOP1*, *BOP*, and C-type genes (*AGAMOUS* and *SEPALLATA*) are related to the changes in the nectary structures of antisense *BcNS* transgenic plants. The results of such study could provide important clues to clarify the molecular mechanism in Chinese cabbage.

## Conclusion

In this study, 34 MtN3/saliva/SWEETs genes were identified in *B. rapa*. We found that the full lengths of the coding regions ranged from 387 bp to 951 bp and encoded 128 amino acids to 316 amino acids. The Ka/Ks ratio of orthologous SWEET genes between *B. rapa* and *A. thaliana* implies that the genes suffered strong purifying selection for retention. Ks values concentrated location between 0.30 and 0.40, after calculating, *B. rapa* diverged from *A. thaliana* at around 10 MYA to 16 MYA. qPCR results indicated that the MtN3/saliva/SWEETs genes family may be involved in the reproductive developmental process of Chinese cabbage. Our study shows that *BcNS* play an important role in the development of the floral nectary in plant.

## Additional files

Additional file 1: **Table S1.** The MtN3/saliva domain sequences of *A. thaliana*. (XLSX 27 kb)

Additional file 2: **Table S2.** Primer sequences for qRT-PCR analysis. (XLSX 11 kb)

Additional file 3: **Figure S1.** Copy number changes in the *B. rapa* and *Arabidopsis* MtN3/saliva/SWEET genes in Clades A–D. The numbers in ellipses and rectangles represent the numbers of MtN3/saliva/SWEET genes in the extant and ancestral species, respectively. The numbers on the branches with plus and minus symbols represent the numbers of gene gains and losses, respectively. (TIFF 953 kb)

Additional file 4: **Figure S2.** Multiple alignment analysis of the MtN3/saliva/SWEET gene family in *B. rapas.* The black box represent the MtN3/saliva/SWEETs domain. The thick lines represent the transmembrane domain. The thick gray line represent the conservative intracellular region. (TIFF 5777 kb)

Additional file 5: **Table S3.** Putative *cis*-elements in promoter region of the 34 *MtN3/saliva/SWEETs* genes in *B. rapa. (XLSX 14 kb)*

Additional file 6: **Table S4.** Secondary structural prediction of the*MtN3/saliva/SWEETs* proteins of *B. rapa. (XLSX 52 kb)*

Additional file 7: **Table S5.** Hydrophobic character prediction based on the Kyte–Doolittle scale for the deduction of *MtN3/saliva/SWEETs* proteins in *B. rapa. (XLSX 59 kb)*

Additional file 8: **Table S6.** Protein subcellular localization prediction of *MtN3/saliva/SWEETs* proteins in *B. rapa. (XLSX 11 kb)*

Additional file 9: **Figure S3.** WebLogo of the most conserved consensus motifs of the amino acids of *B. rapa. (TIFF 1081 kb)*

Additional file 10: **Figure S4.** Prediction of transmembrane helices in Bra000116. (GIF 11 kb)

Additional file 11: **Figure S5.** Obtained transgenic plantlets of *BcNS*. A: Sown seeds; B: seedling at 4–5 days; C: cotyledon–hypocotyl explants during preculture; D–E: cotyledon–hypocotyl explants during differentiation; F: grown seedling; G–H: roots induced from the Hyg^R^ seedling; and I: regenerated plants transferred to the plot. (TIFF 13426 kb)

Additional file 12: **Figure S6.** Positive detection of transgenic Chinese cabbage (*B. campestris* ssp. *chinensis* var. *parachinensis*). A, PCR amplification detection. Lane 1, marker; Lane 2, amplification results of the negative control, water; Lane 3, amplification results of the positive control, 35S-pCAM1BIA1301; Lanes 4–8, amplification results of the positive control, 35S-pCAM1IA1301; Lanes 9–19 amplification results of the 35 s-*BcNS* transformants. B and C, X-Gluc histochemical staining detection of calluses and leaves. D, Fluorogenic quantitative PCR detection; WT, wild type; CK, negative control; A-12, A-13, and A-20, antisense expression plant. (TIFF 1610 kb)

Additional file 13: **Figure S7.** Morphology observation of the flowers of *B. rapa* ssp. *chinensis* var. *parachinesis* transgenic plants. A–D are wild types, E–H are pCAMBIA transgenic plants, and I–L are pCAMBIA*-BcNS* transgenic plants. The arrows refer to the nectaries. (TIFF 1003 kb)

## Abbreviations

*BcNS*: *Brassica* Nectary and Stamen
F: Flower
GSDS: Gene Structure Display Server
L: Leave
LB: large bud
*MtN3*: *Medicago truncatula* Nodulin3
NCBI: National Center for Biotechnology Information
Nt: nectary
Pe: petal
Pi: pistil
qRT-PCR: Quantitative reverse-transcription PCR
R: root
SB: small bud
Se: sepal
Si: Silique
St: stamen
St: stem
WGT: whole-genome triplication

## References

[1] Saier MH, Tran CV, Barabote RD (2006). TCDB: the transporter classification database for membrane transport protein analyses and information. Nucleic Acids Res.

[2] Hamada M, Wada S, Kobayashi K, Satoh N (2005). *Ci-Rga*, a gene encoding an MtN3/saliva family transmembrane protein, is essential for tissue differentiation during embryogenesis of the ascidian *Ciona intestinalis*. Differentiation.

[3] Gamas P, de Carvalho-Niebel F, Lescure N, Cullimore JV (1996). Use of a subtractive hybridization approach to identify new *Medicago truncatula* genes induced during root nodule development. Mol Plant-Microbe Interact.

[4] Artero RD, Terol-Alcayde J, Paricio N, Ring J, Bargues M, Torres A (1998). *Saliva*, a new *Drosophila* gene expressed in the embryonic salivary glands with homologues in plants and vertebrates. Mech Dev.

[5] Yuan M, Chu Z, Li X, Xu C, Wang S (2009). Pathogen-induced expressional loss of function is the key factor in race-specific bacterial resistance conferred by a recessive R gene *xa13* in rice. Plant Cell Physiol.

[6] Udvardi MK, Yang LJO, Young S, Day DA (1990). Sugar and amino-acid-transport across symbiotic membranes from soybean nodules. Mol Plant-Microbe Interact.

[7] Yuan M, Chu Z, Li X, Xu C, Wang S (2010). The bacterial pathogen *Xanthomonas oryzae* overcomes rice defenses by regulating host copper redistribution. Plant Cell.

[8] Antony G, Zhou J, Huang S, Li T, Liu B, White F (2010). Rice *xa13* recessive resistance to bacterial blight is defeated by induction of the disease susceptibility gene *Os-11N3*. Plant Cell.

[9] Ge YX, Angenent GC, Wittich PE, Peters J, Franken J, Busscher M (2000). *NEC1*, a novel gene, highly expressed in nectary tissue of *Petunia hybrida*. Plant J.

[10] Ge YX, Angenent GC, Dahlhaus E, Franken J, Peters J, Wullems GJ (2001). Partial silencing of the *NEC1* gene results in early opening of anthers in *Petunia hybrida*. Mol Gen Genomics.

[11] Guan Y, Huang X, Zhu J, Gao J, Zhang H, Yang Z (2008). *RUPTURED POLLEN GRAIN1*, a member of the MtN3/saliva gene family, is crucial for exine pattern formation and cell integrity of microspores in Arabidopsis. Plant Physiol.

[12] Seo PJ, Park JM, Kang SK, Kim SG, Park CM (2011). An Arabidopsis senescence-associated protein SAG29 regulates cell viability under high salinity. Planta.

[13] Chu Z, Yuan M, Yao L, Ge X, Yuan B, Xu C (2006). Promoter mutations of an essential gene for pollen development result in disease resistance in rice. Genes Dev.

[14] John LB, David RS (1999). *CRABS CLAW*, a gene that regulates carpel and nectary development in Arabidopsis, encodes a novel protein with zinc finger and helix-loop-helix domains. Development.

[15] McKim SM, Stenvik GE, Butenko MA, Kristiansen W, Cho SK, Hepworth SR (2008). The *BLADE-ON-PETIOLE* genes are essential for abscission zone formation in Arabidopsis. Development.

[16] Ruhlmann JM, Kram BW, Carter CJ (2010). *CELL WALL INVERTASE 4* is required for nectar production in *Arabidopsis*. J Exp Bot.

[17] Kram BW, Xu WW, Carter CJ (2009). Uncovering the *Arabidopsis thaliana* nectary transcriptome: investigation of differential gene expression in floral nectariferous tissues. BMC Plant Biol.

[18] Hampton M, Xu WW, Kram BW, Chambers EM, Ehrnriter JS, Gralewski JH (2010). Identification of differential gene expression in *Brassica rapa* nectaries through expressed sequence tag analysis. PLoS One.

[19] Davis AR, Peterson RL, Shuel RW (1986). Anatomy and vasculature of the floral nectaries of *Brassica napus* (Brassicaceae). Can J Botany-Revue Canadienne de Botanique.

[20] Voorrips RE (2002). MapChart: software for the graphical presentation of linkage maps and QTLs. J Hered.

[21] Dung Tien L, Nishiyama R, Watanabe Y, Vankova R, Tanaka M, Seki M (2012). Identification and expression analysis of cytokinin metabolic genes in soybean under normal and drought conditions in relation to cytokinin levels. PLoS One.

[22] Liu Z, Lv Y, Zhang M, Liu Y, Kong L, Zou M (2013). Identification, expression, and comparative genomic analysis of the *IPT* and *CKX* gene families in Chinese cabbage (*Brassica rapa* ssp. *pekinensis*). BMC Genomics.

[23] Thompson JD, Higgins DG, Gibson TJ (1994). Clustal W: improving the sensitivity of progressive multiple sequence alignment through sequence weighting, position-specific gap penalties and weight matrix choice. Nucleic Acids Res.

[24] Saitou N, Nei M (1987). The neighbor-joining method - a new method for reconstructing phylogenetic trees. Mol Biol Evol.

[25] Koch MA, Haubold B, Mitchell-Olds T (2000). Comparative evolutionary analysis of chalcone synthase and alcohol dehydrogenase loci in *Arabidopsis*, *Arabis*, and related genera (Brassicaceae). Mol Biol Evol.

[26] Bailey TL, Boden M, Buske FA, Frith M, Grant CE, Clementi L (2009). MEME SUITE: tools for motif discovery and searching. Nucleic Acids Res.

[27] Erik L, von Gunnar H, Anders K (1998). A hidden Markov model for predicting transmembrane helices in protein sequences. Conf Intell Syst Mol Biol.

[28] Kyte J, Doolittle RF (1982). A simple method for displaying the hydropathic character of a protein. J Mol Biol.

[29] Gasteiger E, Gattiker A, Hoogland C, Ivanyi I, Appel RD, Bairoch A (2003). ExPASy: the proteomics server for in-depth protein knowledge and analysis. Nucleic Acids Res.

[30] Lescot M, Dehais P, Thijs G, Marchal K, Moreau Y, Van de Peer Y (2002). PlantCARE, a database of plant cis-acting regulatory elements and a portal to tools for in silico analysis of promoter sequences. Nucleic Acids Res.

[31] Livak KJ, Schmittgen TD (2001). Analysis of relative gene expression data using real-time quantitative PCR and the 2-[Delta][Delta] CT method. Methods.

[32] Huang L, Cao J, Zhang A, Ye Y, Zhang Y, Liu T (2009). The polygalacturonase gene *BcMF2* from *Brassica campestris* is associated with intine development. J Exp Bot.

[33] Han J, Jiang J (2015). Genome-wide analysis of SWEET gene family in *Arabidopsis thaliana*, *Oryza sativa* and *Lycopersicum esculentum*. Mol Plant Breed.

[34] Swanson WJ, Zhang ZH, Wolfner MF, Aquadro CF (2001). Positive Darwinian selection drives the evolution of several female reproductive proteins in mammals. Proc Natl Acad Sci U S A.

[35] Guermeur Y, Geourjon C, Gallinari P, Deleage G (1999). Improved performance in protein secondary structure prediction by inhomogeneous score combination. Bioinformatics.

[36] Du H, Yang C, Ding G, Shi L, Xu F (2017). Genome-wide identification and characterization of SPX domain-containing members and their responses to phosphate deficiency in *Brassica napus*. Front Plant Sci.

[37] Liu X, Lu Y, Yana M, Sun D, Hu X, Liu S (2016). Genome-wide identification, localization, and expression analysis of Proanthocyanidin-associated genes in *Brassica*. Front Plant Sci.

[38] Yuan M, Zhao J, Huang R, Li X, Xiao J, Wang SP (2014). Rice MtN3/saliva/SWEET gene family: evolution, expression profiling, and sugar transport. J Integr Plant Biol.

[39] Feng C, Han J, Han X, Jiang J (2015). Genome-wide identification, phylogeny, and expression analysis of the SWEET gene family in tomato. Gene.

[40] Beilstein MA, Nagalingum NS, Clements MD, Manchester SR, Mathews S (2010). Dated molecular phylogenies indicate a Miocene origin for *Arabidopsis thaliana*. Proc Natl Acad Sci U S A..

[41] Yang Y, Lai K, Tai P, Li W (1999). Rates of nucleotide substitution in angiosperm mitochondrial DNA sequences and dates of divergence between *Brassica* and other angiosperm lineages. J Mol Evol.

[42] Town CD, Cheung F, Maiti R, Crabtree J, Haas BJ, Wortman JR (2006). Comparative genomics of *Brassica oleracea* and *Arabidopsis thaliana* reveal gene loss, fragmentation, and dispersal after polyploidy. Plant Cell.

[43] Bowers JE, Chapman BA, Rong JK, Paterson AH (2003). Unravelling angiosperm genome evolution by phylogenetic analysis of chromosomal duplication events. Nature.

[44] Ziolkowski PA, Kaczmarek M, Babula D, Sadowski J (2006). Genome evolution in *Arabidopsis/Brassica*: conservation and divergence of ancient rearranged segments and their breakpoints. Plant J.

[45] Chen L, Hou B, Lalonde S, Takanaga H, Hartung ML, Qu X (2010). Sugar transporters for intercellular exchange and nutrition of pathogens. Nature.

[46] Ferrari S, Galletti R, Denoux C, De Lorenzo G, Ausubel FM, Dewdney J (2007). Resistance to *Botrytis cinerea* induced in Arabidopsis by elicitors is independent of salicylic acid, ethylene, or jasmonate signaling but requires *PHYTOALEXIN DEFICIENT3*. Plant Physiol.

[47] Sun M, Huang X, Yang J, Guan Y, Yang Z (2013). Arabidopsis RPG1 is important for primexine deposition and functions redundantly with RPG2 for plant fertility at the late reproductive stage. Plant Reprodn.

[48] Kobayashi T, Kobayashi E, Sato S, Hotta Y, Miyajima N, Tanaka A (1994). Characterization of cDNAs induced in meiotic prophase in lily microsporocytes. DNA Res.

[49] Salts Y, Sobolev I, Chmelnitsky I, Shabtai S, Barg R (2005). Genomic structure and expression of *Lestd1*, a seven-transmembrane-domain protein-encoding gene specifically expressed in tomato pollen. Israel J Plant Sci.

[50] Allen AM, Thorogood CJ, Hegarty MJ, Lexer C, Hiscock SJ (2011). Pollen-pistil interactions and self-incompatibility in the Asteraceae: new insights from studies of *Senecio squalidus* (Oxford ragwort). Ann Bot.

[51] Quiapim AC, Brito MS, Bernardes LAS, DaSilva I, Malavazi I, DePaoli HC (2009). Analysis of the Nicotiana Tabacum Stigma/style transcriptome reveals gene expression differences between wet and dry stigma species. Plant Physiol.

[52] Kay S, Hahn S, Marois E, Wieduwild R, Bonas U (2009). Detailed analysis of the DNA recognition motifs of the *Xanthomonas* type III effectors AvrBs3 and AvrBs3 Delta rep16. Plant J.

[53] Li W, Yang J, Gu X (2005). Expression divergence between duplicate genes. Trends Genet.

[54] Zhang F, Chen S, Zhao X, Bai X, Du Y (2009). Construction of NtSKP1-GFP plant expression vector and subcellular location. Acat Agriculturae Boreali-Occidentalis Sinica.

[55] Yang B, Sugio A, White FF (2006). *Os8N3* is a host disease-susceptibility gene for bacterial blight of rice. Proc Natl Acad Sci U S A.

[56] Macukanovic-Jocic MP, Rancic DV, Stevanovic ZPD (2007). Floral nectaries of basil (*Ocimum basilicum*): morphology, anatomy and possible mode of secretion. S Afr J Bot.

[57] Pyke GH (1991). What does it cost a plant to produce flower nectar. Nature.

[58] Bender RL, Fekete ML, Klinkenberg PM, Hampton M, Bauer B, Malecha M (2013). *PIN6* is required for nectary auxin response and short stamen development. Plant J.

[59] Radhika V, Kost C, Boland W, Heil M (2010). The role of jasmonates in floral nectar secretion. PLoS One.

[60] Wiesen LB, Bender RL, Paradis T, Larson A, Perera M, Nikolau BJ (2016). A role for GIBBERELLIN 2-OXIDASE6 and gibberellins in regulating nectar production. Mol Plant.

[61] Baum SF, Eshed Y, Bowman JL (2001). The Arabidopsis nectary is an ABC-independent floral structure. Development.

[62] Lee JY, Baum SF, Alvarez J, Patel A, Chitwood DH, Bowman JL (2005). Activation of *CRABS CLAW* in the nectaries and carpels of Arabidopsis. Plant Cell.

