# Supplementary figures and images for: Genome-wide identification, phylogeny, evolution, and expression patterns of MtN3/saliva/SWEET genes and functional analysis of BcNS in Brassica rapa
Source: BMC Genomics. 2018 Mar 2;19:174. doi: 10.1186/s12864-018-4554-8 (PMC5834901; doi:10.1186/s12864-018-4554-8)

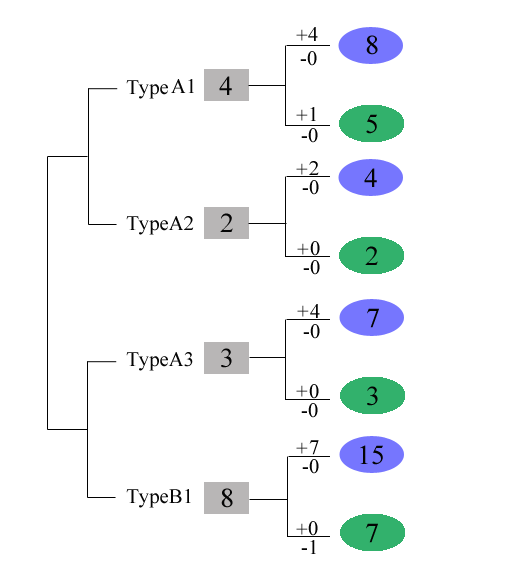

Supplement: Supplementary file 3 — Figure S1. Copy number changes in the B. rapa and Arabidopsis MtN3/saliva/SWEET genes in Clades A–D. The numbers in ellipses and rectangles represent the numbers of MtN3/saliva/SWEET genes in the extant and ancestral species, respectively. The numbers on the branches with plus and minus symbols represent the numbers of gene gains and losses, respectively. (TIFF 953 kb) [file 12864_2018_4554_MOESM3_ESM.tif]

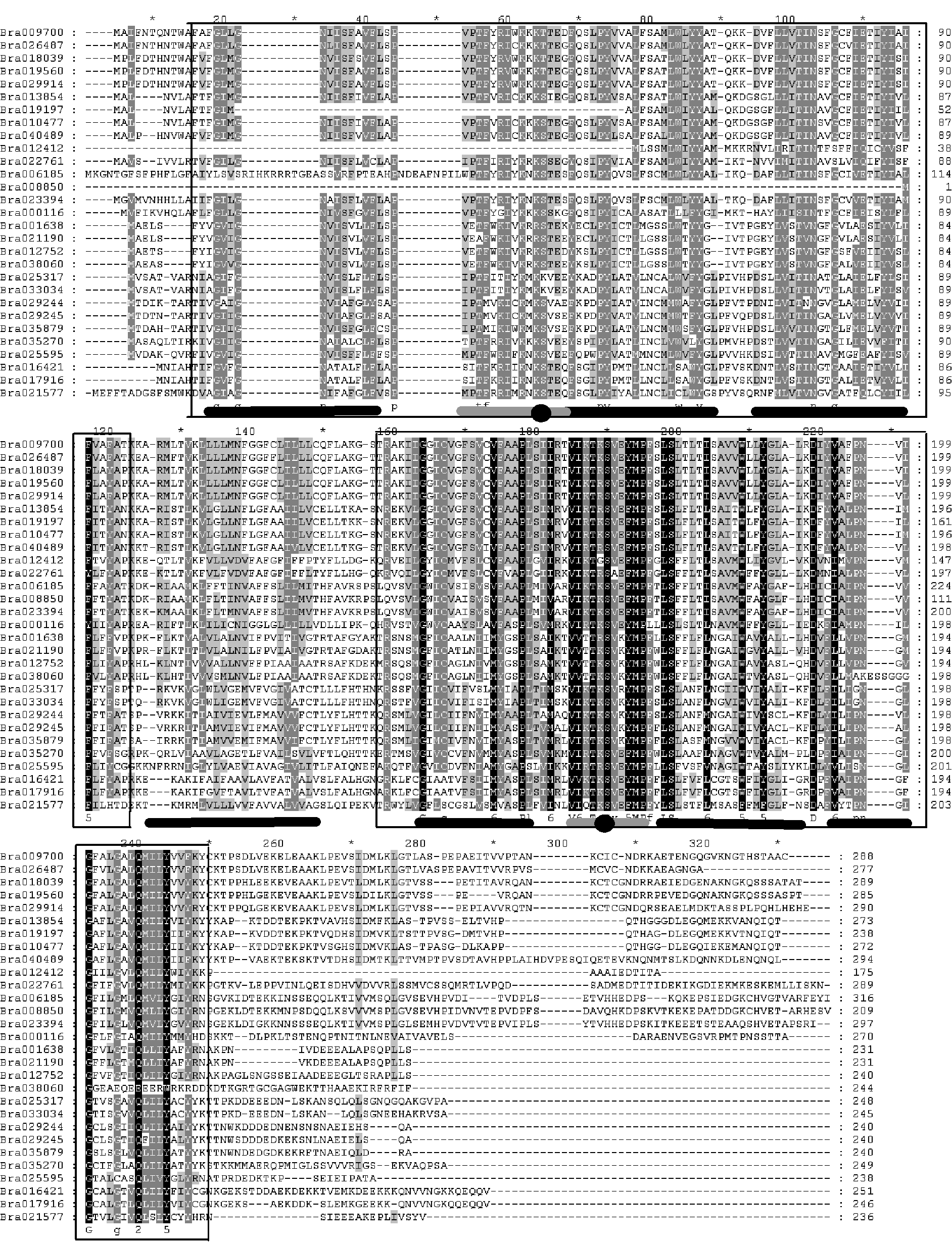

Supplement: Supplementary file 4 — Figure S2. Multiple alignment analysis of the MtN3/saliva/SWEET gene family in B. rapas. The black box represent the MtN3/saliva/SWEETs domain. The thick lines represent the transmembrane domain. The thick gray line represent the conservative intracellular region. (TIFF 5777 kb) [file 12864_2018_4554_MOESM4_ESM.tif]

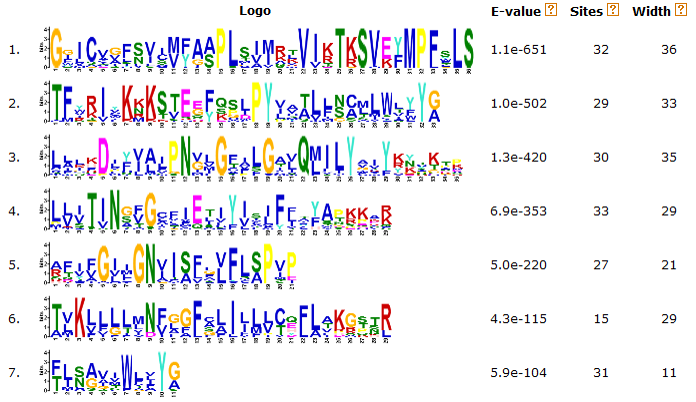

Supplement: Supplementary file 9 — Figure S3. WebLogo of the most conserved consensus motifs of the amino acids of B. rapa. (TIFF 1081 kb) [file 12864_2018_4554_MOESM9_ESM.tif]

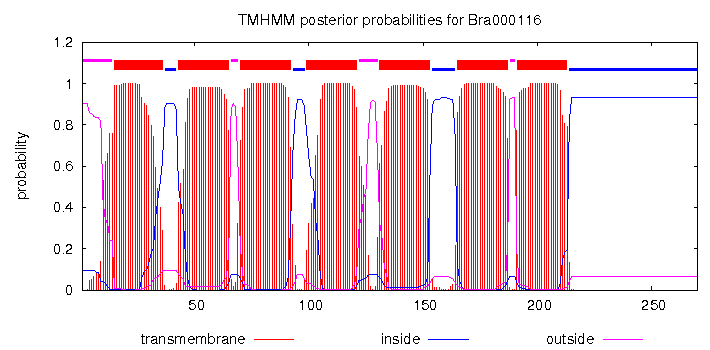

Supplement: Supplementary file 10 — Figure S4. Prediction of transmembrane helices in Bra000116. (GIF 11 kb) [file 12864_2018_4554_MOESM10_ESM.gif]

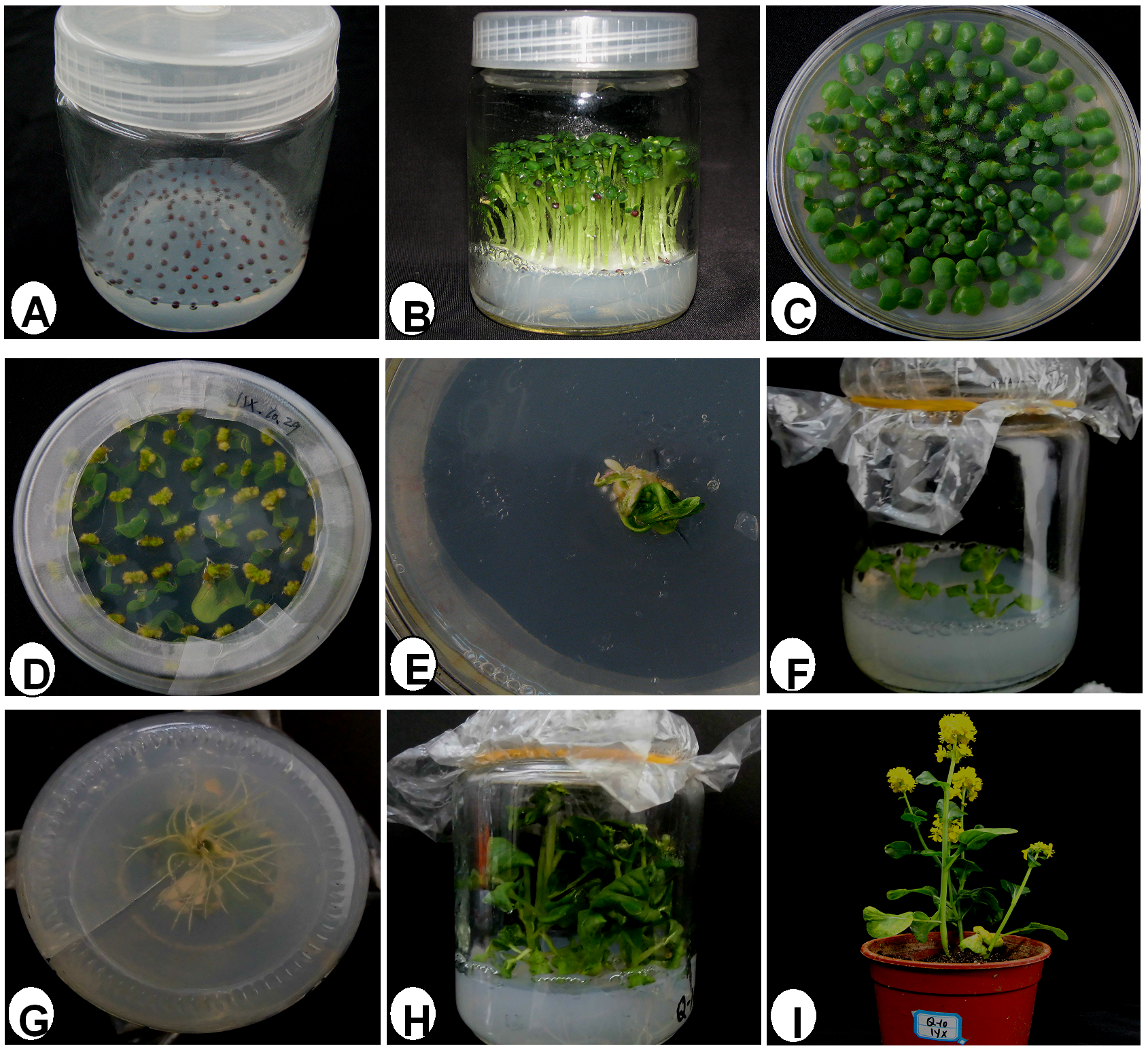

Supplement: Supplementary file 11 — Figure S5. Obtained transgenic plantlets of BcNS. A: Sown seeds; B: seedling at 4–5 days; C: cotyledon–hypocotyl explants during preculture; D–E: cotyledon–hypocotyl explants during differentiation; F: grown seedling; G–H: roots induced from the HygR seedling; and I: regenerated plants transferred to the plot. (TIFF 13426 kb) [file 12864_2018_4554_MOESM11_ESM.tif]

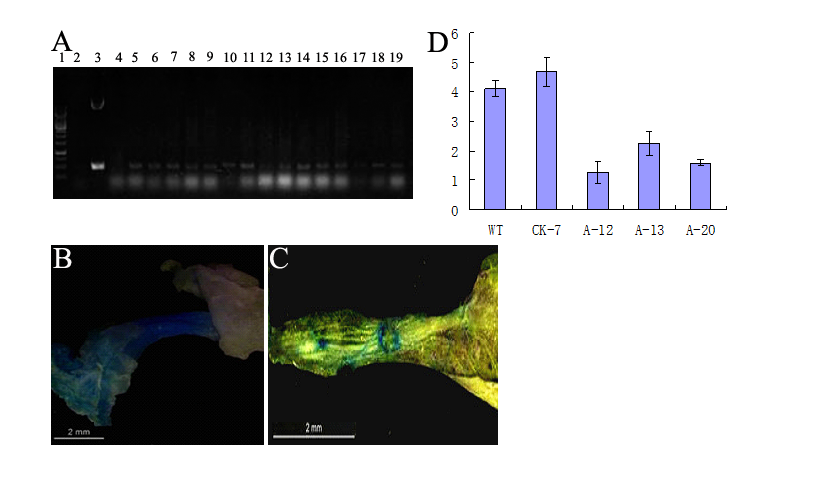

Supplement: Supplementary file 12 — Figure S6. Positive detection of transgenic Chinese cabbage (B. campestris ssp. chinensis var. parachinensis). A, PCR amplification detection. Lane 1, marker; Lane 2, amplification results of the negative control, water; Lane 3, amplification results of the positive control, 35S-pCAM1BIA1301; Lanes 4–8, amplification results of the positive control, 35S-pCAM1IA1301; Lanes 9–19 amplification results of the 35 s-BcNS transformants. B and C, X-Gluc histochemical staining detection of calluses and leaves. D, Fluorogenic quantitative PCR detection; WT, wild type; CK, negative control; A-12, A-13, and A-20, antisense expression plant. (TIFF 1610 kb) [file 12864_2018_4554_MOESM12_ESM.tif]

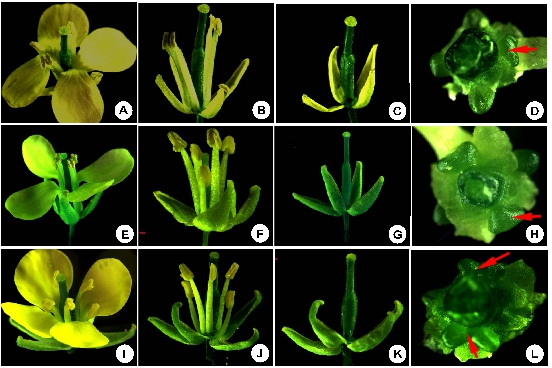

Supplement: Supplementary file 13 — Figure S7. Morphology observation of the flowers of B. rapa ssp. chinensis var. parachinesis transgenic plants. A–D are wild types, E–H are pCAMBIA transgenic plants, and I–L are pCAMBIA-BcNS transgenic plants. The arrows refer to the nectaries. (TIFF 1003 kb) [file 12864_2018_4554_MOESM13_ESM.tif]
